# Enhancement of quasi-static compression strength for aluminum closed cell foam blocks shielded by aluminum tubes

**DOI:** 10.1038/s41598-023-33750-7

**Published:** 2023-04-28

**Authors:** Mohamed H. Dadoura, Ahmed Ismail Zaky Farahat, M. R. Taha, Ramadan N. Elshaer

**Affiliations:** 1 Helwan University, Cairo, Egypt; 2 Central Metallurgical Research and Development Institute, Cairo, Egypt; 3 Faculty of Engineering, Cairo University, Cairo, Egypt; 4 Tabbin Institute for Metallurgical Studies, Cairo, Egypt

**Keywords:** Engineering, Materials science

## Abstract

Aluminum closed cell foam blocks are created with a volume of 1 inch^3^ which consist of aluminum foam parts shielded with part of aluminum tube and in some types reinforced with inner aluminum tubes. Blocks have been made to overcome some existing problems in metallic foam used to protect some applications parts from impacts as a sacrificial part. Metallic foam has three main categories sandwich panels, filled tubes and corrugated sheets. Quasi-static compression tests have been applied on 12 blocks with different shapes and compared with pure aluminum foam blocks as a reference. Results display the enhancement of mechanical properties of blocks like yield strength (S_Y_), crushing strength (S_c_) and densification strength (S_d_), compression at strain 70%, as well as absorbed energy (area of compression under the curve). The highest value for yield strength (5.87 MPa) was registered for Finger phalanxes cube block (FP—0.1 Sq.). While the highest value for densification strength (21.7 MPa) was registered for spine cylinder block (SV8—0.17 C25). The registered results for samples apparent the highest value for energy dissipation density (E_dd_) is 40.52 J/in^3^ (91% enhancement) and crushing strength (8.61 MPa) was registered for Finger phalanx cylinder block (FP—0.17 C25). The lowest value for E_dd_ is 14.16 J/in^3^ (less than pure aluminum foam block value by 33%), S_Y_ = 0.42 MPa, Sc = 3.21 MPa, and S_d_ = 4.46 MPa, registered for thin wall Ear canal cylinder block (EC8—0.075 C26.5). Best mechanical properties had been achieved for Finger phalanx cylinder block (FP—0.17 C25) and spine cylinder block (SV8—0.17 C25).

## Introduction

Aluminum (Al) foam was fabricated in the middle of the last century. It was used in many applications such as supporting some parts of cars and containers to absorb shocks and enhance the isolation of sound and heat. Al closed cell foam (ACCF) is considered an expendable material in applications where it works as a sacrificial part that absorbs energy to protect parts or machines from hard impacts. Despite there are several forms of it like sandwich panels, filled tubes, and corrugated sheets used in industrial applications, it still faces some challenges like high cost of production and the high cost of casting parts or filling tubes, after impacts defective parts or sheets are replaced by new one as usual if possible, where it can’t be repaired which lead to the high cost of maintenance (i.e. Al foam parts when expose to impact bending stress it will be deformed and must be replaced totally). Al foam was used in a lot of applications from lightweight building walls and roofs (i.e., steel Al sandwich (SAS) and Al foam sandwich panels (AFS)) to crashworthiness in automobiles.

**Figure 1:**
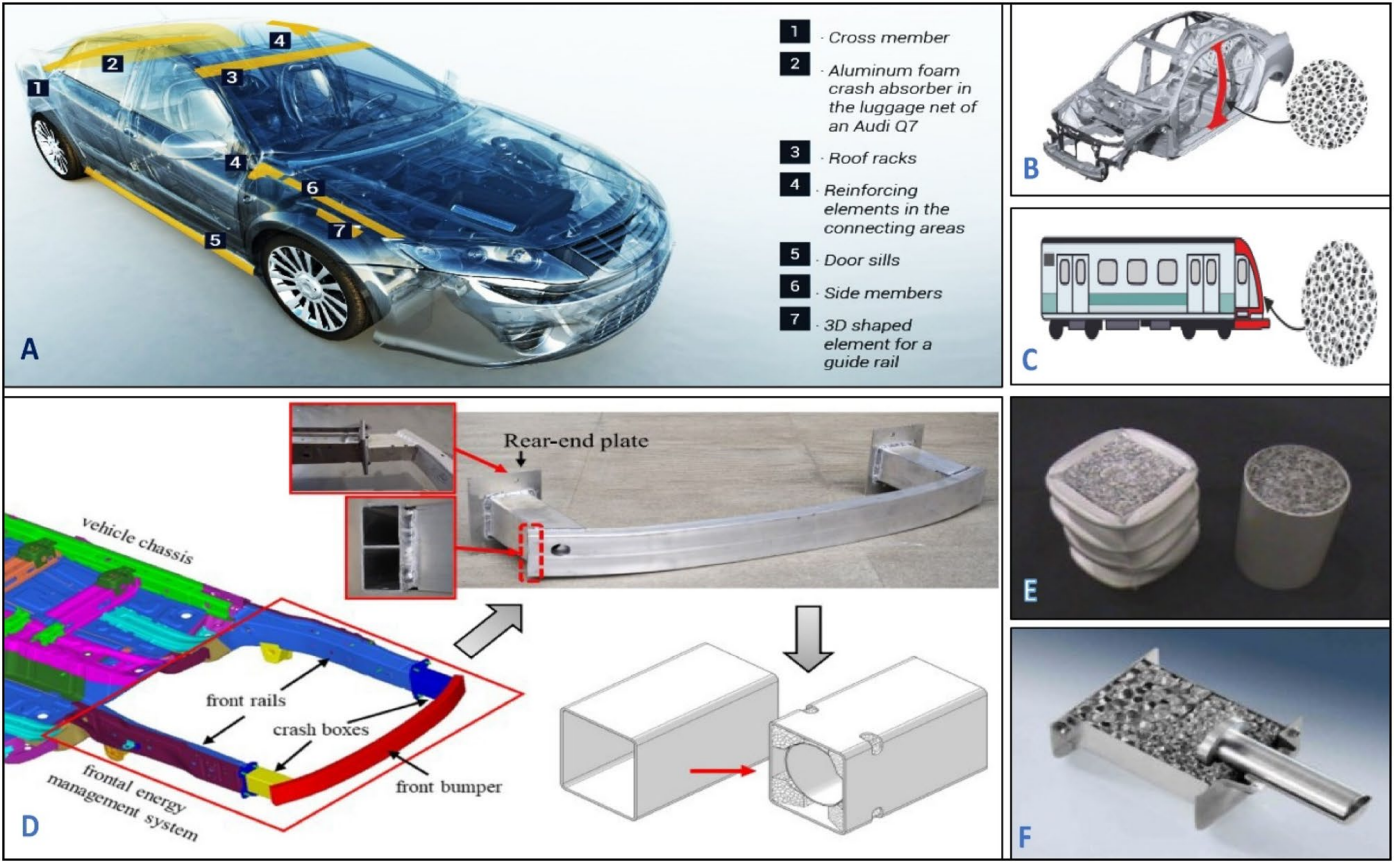
Some applications used of Al foam (**a**) Shielded parts by foam in automotive^1^, (**b**) Shielding car frame by foam, (**c**) Crumble zone shielded by foam in trains, (**d**) Crash boxes of car front bumper^2^, (**e**) Cylindrical and polygonal crash boxes, and (**f**) Crash boxes with two foam parts with different cell sizes^3^.

Figure 1 shows some applications for aluminum foam parts that are used in shielding automotive frames against impacts also, trains used foam in crumple zones where it has lightweight and ability to absorb high energy during impact. The most important applications of metallic foam are crash boxes which shield the front bumper of cars. The most common type is a cylindrical canister or polygonal canister filled with foam as appears in Fig. 1e. Many shapes and models have been made for this box with different techniques like adding two parts of foam with different cell sizes where foam with big size face impact then put the one with less size (Fig. 1f) to improve energy absorption through gradual the shock absorption. The challenge still exists is the cost of maintaining the impacted parts plus the high cost of making this shield with controlled limited dimensions.

The solid foam material is classified into (1) Natural materials like human and animal bones, cancellous bone, cuttlefish bone and coral. (2) artificial materials like Steel foam, Aluminum foam and some cellular polymers^4^. ACCFBs have been invented from traditional components to overcome the cost of fabrication foam for nonuniform shapes and the cost of maintenance for uniform shapes. Bones are the optimum design created by great creators which combined compact bone and spongy bones. It can bear stresses and absorb high energy during motion the awesome thing is accurate distribution for dimensions of bones which are considered as a group of blocks that have limited sizes and this makes every part able to apply its function in easy way.

**Figure 2:**
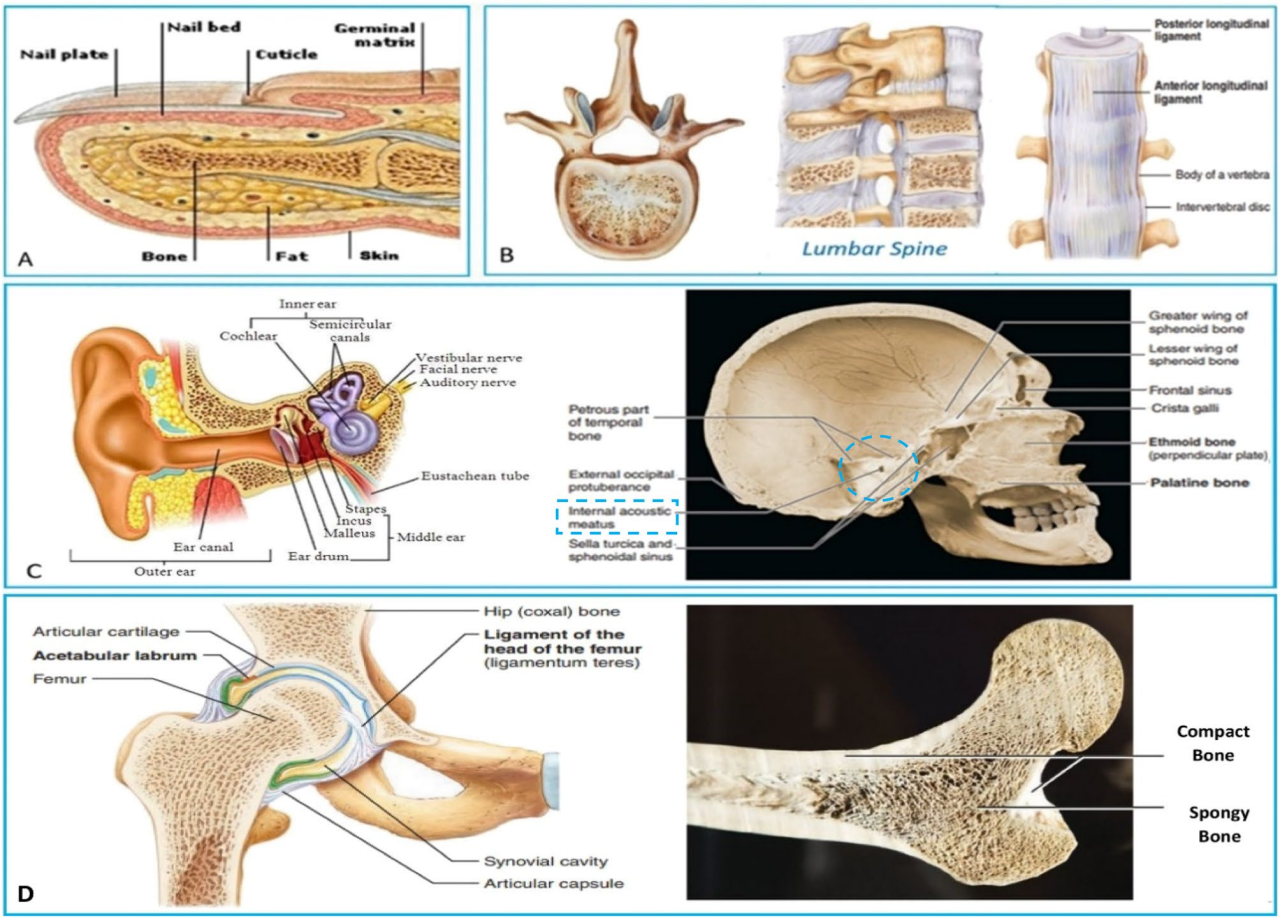
(**a**) Hand anatomy, phalanx bone formation^5^, (**b**) Spine (Lumbar Vertebrae)^6^, (**c**) Ear canal structure and components^6,7^, and (**d**) Femur bone front section^7^.

Three categories have been simulated by aluminum foam with different shapes and dimensions distribution for the same volume (1) Finger phalanx blocks where in real cases when collecting finger phalanx in punch position bones will be able to increase its energy absorption by 4 to 5 times. (2) Spine blocks which able to save expensive wire or cable and can control bending angle by putting flexible spacers between spine bones which simulate discs in real. Spine shields spinal cord from impacts despite its flexible motion in body. (3) Ear canal blocks are able to shield expensive cables too like spine blocks but with equal conditions surrounding their surface. Figure 2 shows the finger phalanx bone formation, lumbar spine photo and cross section, ear canal placed in cranium and cross section for it and femur bone cross section. From the mechanical point of view the muscles, fats, and skin are considered damping materials where it helps in reducing the impact of stress on bones.

Problems could be concluded in high cost of foam production, maintenance and high thermal isolation which is harmful to some applications. So, Blocks are made to simulate some bones ideas in human skeleton like bones of finger phalanx, spine (vertebrae) and ear canal bones in skull. The idea of this research came from meditation on skeleton bones where bones are created from calcium foam (spongy bone) shielded with a hard calcium layer (compact bone) and sometimes bones are reinforced with compact bones inside their part according to function and applied stresses which created to bear it like ear canal bones and spine bones.

## Experimental work

The advantages of Al foam are high energy absorption through plastic deformation excellent vibration dampening, heat and sound isolation for a density bigger than 400 kg/m^3^ and it can be recycled. The advantages of ACCFBs are: availability of tubes in traditional market by different, materials, sizes and thicknesses also, foam can be chosen according to its type, density and cell size. ACCFBs consist of aluminum foam which simulates spongy bone, and small parts of Al tubes to simulate compact bone shields. Al tubes have rectangular and circular shapes for outer shields and inner reinforcement circular tubes with small diameters tubes of 8 and 10 mm.

Aluminum Foam is a composite material defined as a special case of porous metals where a solid foam originates from a liquid foam in which gas bubbles are finely dispersed in a liquid with semi-equal sizes. Porous metal’s relative density (*P*_rel_) should not be greater than 70%. Metallic foam in common can arrive at 30%^8^. Note that a higher density of the foam means stiffness increases and energy absorption ability will be decreased.

1 $$ \uprho_{{{\text{rel}} = }} \uprho_{{{\text{foam}} }} /\uprho_{{\text{base metal}}} $$

**Figure 3:**
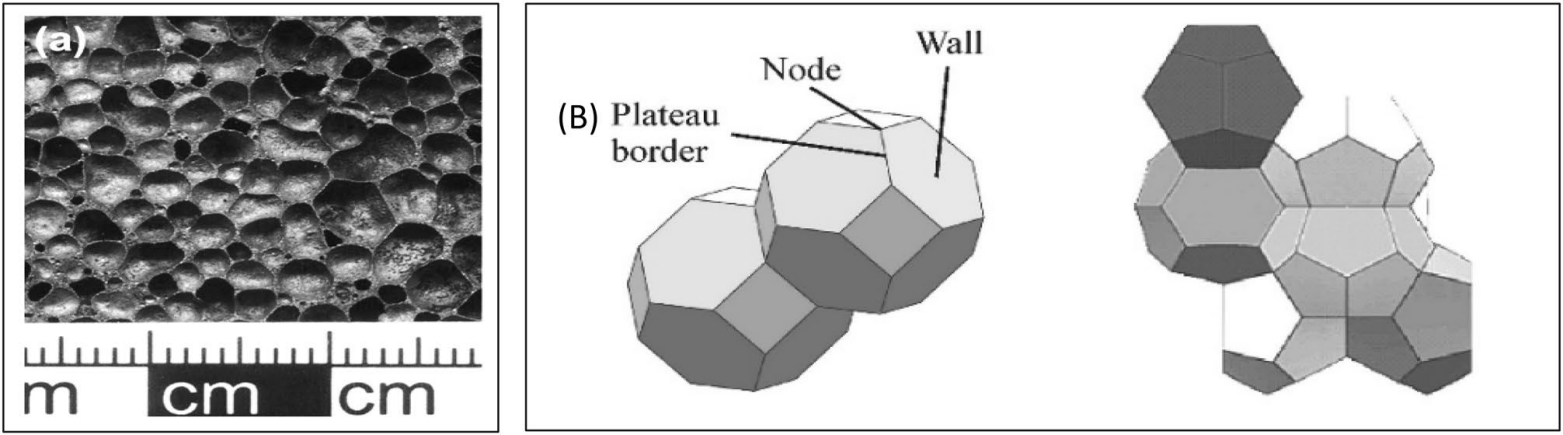
(**a**) Closed-cell Al foam produced by the precursor route with TiH_2_^10^ and (**b**) Component of foam cells.

Foam is defined an as amorphous alloy according to its crystal structure where the atoms have no opportunity to form a crystalline lattice and solidify in a disordered manner due to foam bubble formation. Foam cells consist of cell walls, Plateau borders, and nodes (see Fig. 3). *Cell wall:* separates two gas bubbles over a length of about the bubble diameter and shows a curvature that is much smaller than the mean curvature of the two bubbles. Generally, the mean cell wall thickness is much smaller than the bubble diameter. *Plateau borders:* defined as the intersections of the walls. *Nodes:* are junctions of at least the four Plateau borders, nodes formed when plateau borders are disordered and form a network^9^.

**Figure 4:**
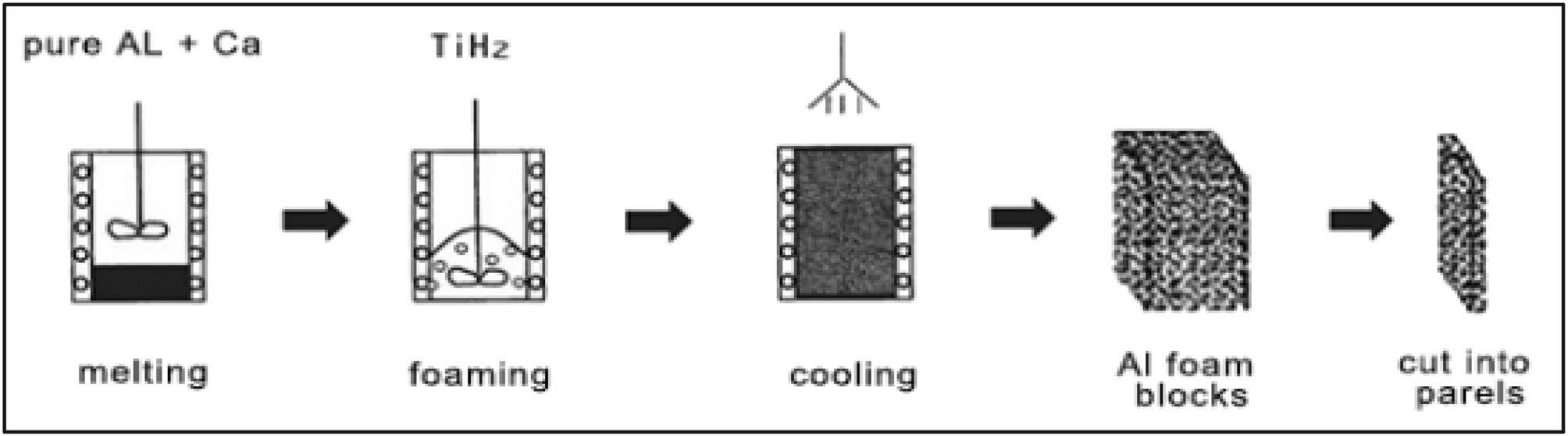
Closed-cell Al foam manufacturing procedures.

The manufacture data and properties of Al foam used in ACCFBs which conclude type, chemical composition (Base material, reinforcement material, precursor), cell size, density, and compressive strength at 70% strain are listed in Table 1. To ensure that all samples have equal properties all blocks have been cut from one sheet of Al foam with a density of 400 kg/m^3^ and wall thickness in a range of 0.2 to 0.23 mm. Figure 4 shows the procedures for producing Al foam by manufacture: Smelting pure aluminum matrix at 680 °C then adding thickening material (1.5 wt% Ca) and mixing it with aluminum melt after that transfer smelting mixture from smelting furnace to foaming furnace for viscosification and foaming process by add foaming agent (1.6 wt% TiH_2_) and mix it with rotating impeller. Melt decomposes under the influence of heat and it releases hydrogen gas. As a result, the foam expands and fills up the mold within 15 to 20 min. After mold arrives to specified cell size cooling process will start with air or water after that slab will be ready to saw according to the required dimensions^11,12^.

**Table 1:** Properties of aluminum foam.

Al foam manufactured and supplied by: CHALCO ALUMINUM FABRICATION – *China*		
Type of foam: Alporas (Closed cell)	Cell size: 4 mm	Wall thickness: 0.22 mm
Chemical composition		
Base material: Al 1050	Void percent: 86%	Relative Density: 14%
Precursor: TiH_2_		
Thickening agent: Ca		
Compressive strength: 8.4 MPa	Strain: 70%	Density: 0.378 g/cm^3^

**Figure 5:**
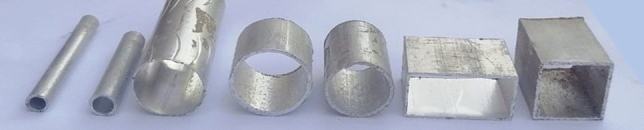
Aluminum 6060 hollow tubes.

Aluminum tube are available in the traditional market dimensions are listed in Table 2. The chemical composition and designation are listed in Table 3 which agreed with standard DIN EN 755-2—AS/NZS 1866^13^. Figure 5 shows the photo of Al tubes that have been used.

**Table 2:** Aluminum tubes dimensions and designation.

Tube cross-section	Square	Rectangular	Circular				
Dimension (mm)	25 × 25	20 × 40	Φ 26.5	Φ 25	Φ 30	Φ 8	Φ 10
Thickness (mm)	1	1	0.75	1.7	1.7	1.2	1.25

**Table 3:** Chemical composition analysis and designation of Aluminum tubes material.

Chemical composition wt%									
Fe	Si	Mn	Cr	Ti	Cu	Mg	Zn	Others	Al
0.196	0.383	0.015	0.020	0.018	0.010	0.425	< 0.001	0.023	98.91
Material designation: DIN EN 755-2—AS/NZS 1866						Grade: Al 6060

**Figure 6:**
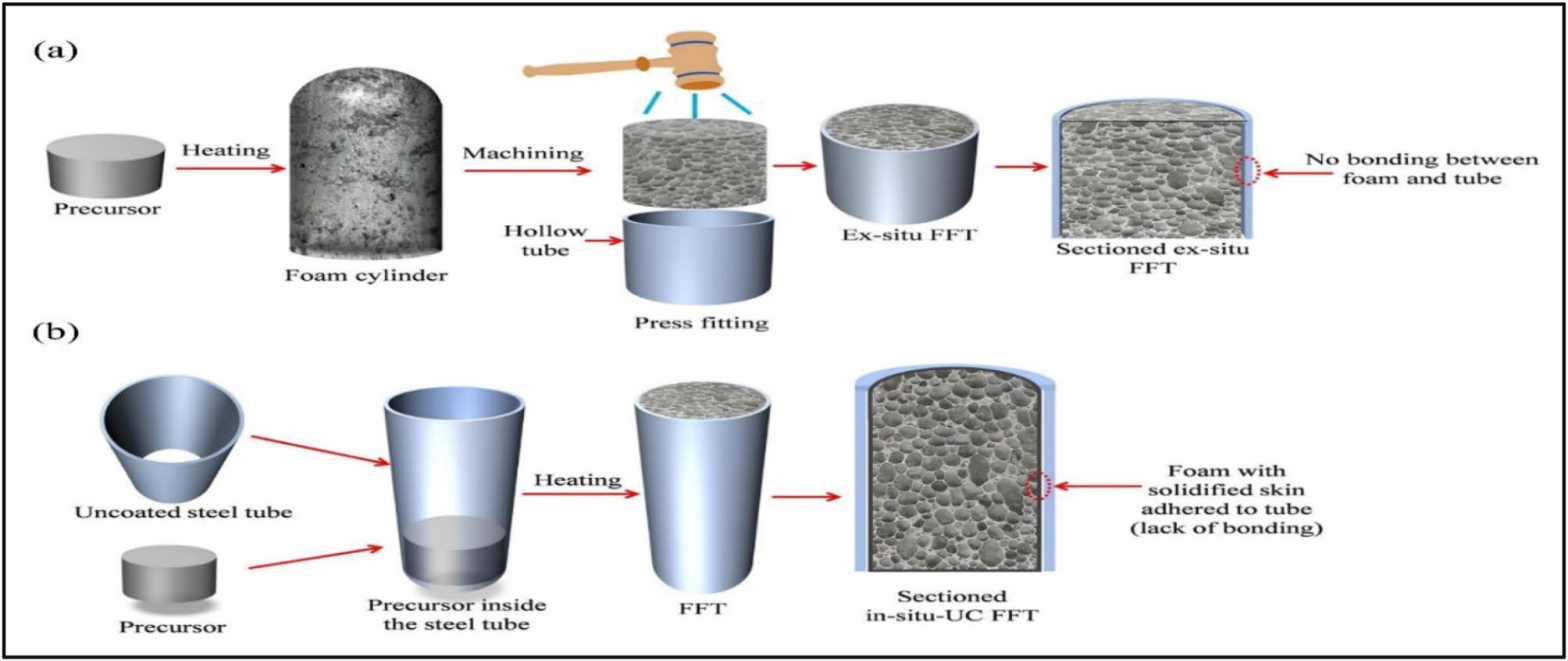
Schematic illustration of the foam filled tube fabrication of (**a**) external situ and (b) internal situ foam filling.

ACCFBs fabricated by machining tools (i.e. Saw, files, drill and rubber hammer) for Al foam and tubes parts the blocks have been assembled according to their designed dimensions and adhesive epoxy metal has been used at the ends of blocks to confirm that parts assembled well together. Actually, there are two methods to make blocks as appears in Fig. 6 the first method is machining both Al foam and tube parts and then assembling them by soft hammering^14^. The second method is building up foam inside tubes profile by mixing first powder metallurgical matrix material, foaming agent (TiH_2_ or ZrH) and additives (Mg, Si, … etc.) then making cold compacting and then hot extruding at about 400–480 °C. The foaming agent thus becomes uniformly distributed and gas-tightly embedded in the metal matrix. The extrusion process is useful in helping to break up the oxide films on the surface of the metal powders, which facilitates consolidation. The product may be considered as a precursor material, itself not far from full density but readily convertible to foam. This conversion is affected by simply heating the precursor to a temperature at which the alloy is liquid. The foaming agent evolves gas, thus creating a foam that is stabilized by very fine oxide particles uniformly distributed throughout the precursor after extrusion. After melting and foaming, the foamed panel is rapidly cooled to prevent collapse of the foamed structure^15^. Although the second method will be producing more strengthen blocks and be cheaper in total cost of fabrication than the first method it is suitable and more reliable for mass production and accurate parts like prosthetics. The first method is easy, general, and gives variety in use where any type of foam can be selected with the required properties^16^, and selection of tubes with different dimensions is easy to be made by common man with limited block numbers according to needs.

**Figure 7:**
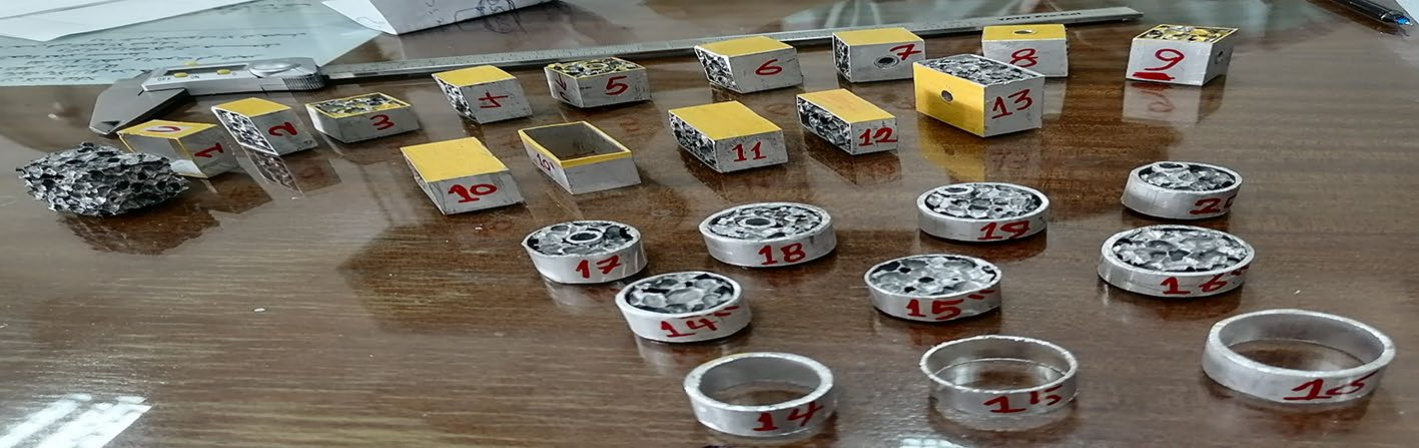
Samples of Aluminium foam Blocks with limited sizes.

ACCFBs twenty samples have been made as appears in Fig. 7. Twelve of them have been selected for applying lateral compression (quasi-static) test and comparing results with pure foam block to specify the enhancement values. The samples simulate four categories of bones: pure foam block, finger phalanxes, spine, and ear canal with different shapes (cube, parallelogram, and cylindrical) as appeared in Table 4. Quasi-static test has been applied and all curve’s data scaled to area cm^2^.

**Table 4:** Pure foam, Finger phalanx, Spine and Ear canal blocks dimensions and codes.

Block type	Block shape	Sample code	Mass (gm)	Vol. at 1 inch^3^ (cm^3^)	Dimensions (mm)							Geometrical properties		
					X	Y_a_	Y_v_	Z	T	${\varnothing }_{in}$	t	A (mm^2^)	Z_*p*_ (mm^3^)	I (mm^4^)
Pure Foam		Pure—Sq	5.91	15.625	25	25	25	25	1	–	–	625	2604.2	32,552
Finger Phalanx blocks		FP—0.1 Sq	11.48	15.625	25	25	25	25	1	–	–	625	2604.2	32,552
		FP—0.1 Pa	11.43	16	40	16	20	20	1	–	–	800	2666.7	26,666
		FP—0.17 C25	15.18	15.707	Φ = 25	25	32	-	1.7	–	–	490.87	1534	19,175
		FP—0.075 C26.5	9.78	15.443	Φ = 26.5	24	28	-	0.75	–	–	551.55	1827	24,208
		FP—0.17 C30	13.60	15.550	Φ = 30	21	22	-	1.7	–	–	706.86	2650.7	39,761
Spine blocks		SV8—0.1 Sq	12.73	14.987	25	25	25	25	1	8	1.2	600.37	2540	31,119
		SV8—0.1 Pa	14	14.724	40	16	20	20	1	8	1.2	750.74	2617.5	25,339
		SV8—0.17 C25	16.80	14.890	Φ = 25	25	32	-	1.7	8	1.2	466.24	1479.9	17,939
		SV8—0.17 C30	14.70	14.988	Φ = 30	21	22	-	1.7	8	1.2	682.23	2669.9	42,245
Ear canal blocks		EC8—0.1 Sq	12.73	14.987	25	25	25	25	1	8	1.2	600.37	2466.2	32,504
		EC8—0.075 C26.5	11.18	14.753	Φ = 26.5	24	28	-	0.75	8	1.2	526.92	1689.1	24,160
		EC8—0.17 C30	14.70	14.988	Φ = 30	21	22	-	1.7	8	1.2	682.23	2512.8	39,712

where X = Block length, Ya = Block length for compression test, Yv = Block length for volume 1 inch^3^, Z = Block height, ϕ = Circular block diameter, T = Shell tube thickness, ϕ_in_ = Inner tube diameter, t = Inner tube thickness, A = Cross section area, I = Moment of area^17,18^, Z_P_ = Section modulus^19^.

Quasi-static compression tests have been applied on the universal testing machine (WDW-300 KN, China). The test velocity was 1 mm/min. Aluminum foam compression test was applied in accordance with standard “*DIN 50,134:2008-10*” of “Testing of metallic materials–Compression test of metallic cellular materials”^20^. Where compressive strain (Ɛ) equals change in length / original length.

2 $$\upvarepsilon \, = \, \Delta {\text{ h }}/{\text{h}} $$

All types of aluminum foam in quasi-static compression tests have plastic collapse regions at strain between 65 and 75%. It is depending on the relative density, cell size, and material composition of the foam matrix. So, all ACCFB categories have been tested at strain 70%. There are two types of energy absorption criteria, which are specific energy absorption capacity (*Es*) and volumetric energy absorption (*E*_*dd*_). *Es* can be defined as the total absorbed energy per unit mass and it is a performance index used in measuring the capacity of a material to absorb energy from an impacting load. It is defined as the ratio of maximum energy that can be dissipated by a unit of foam mass (*m*_*f*_) and *Ea* is described as the potential energy of absorption which is equal to the area under the “stress–strain curve”^21,22^.

3 $$ {\text{Es }} = {\text{ Ea }}/{\text{ m}}_{{\text{f}}} $$

The energy-absorption capacity may also be expressed in terms of the average foam crush strength, (*S*_*c*_), which is defined in foam: stress at which continuous plastic collapse begins. So, over a range of foam deformation. *Es* can be calculated, using the stress–strain curves produced by tests, assuming uniform loading is achieved.

4 $$ {\text{Es }} = \, \left( {{\text{Sc}} \,{\text{d}}} \right) \, /\uprho $$

5 $$ {\text{Ea }} = \, \left( {{\text{Sc}}\, {\text{Vc}}} \right) $$

where V is foam block volume (cm^3^), Vc is compressed volume of foam block (cm^3^), d is Foam deformation, Vc/V (dimensionless), ρ is Density of foam, (gm/cm^3^).

Static energy-dissipation density of foams (*E*_*dd*_) is a useful index to measure aluminum foam's energy absorption properties. This is the maximum energy that a unit volume of foam can absorb due to impact^8^.

6 $$ {\text{E}}_{{{\text{dd}}}} = {\text{ Es}}\,\uprho $$

The volume of ACCFBs have been chosen to be 1 inch^3^ to study the amount of energy absorption due to dimensions redistribution on different geometrical shapes. Some body may have sound say impact test applied on effected area as impact toughness of solid metals or composites (i.e. Charpy test or Izod tests) but in real energy absorption in foam materials defined generally by *E*_*dd*_.

Actually, impact is defined in mechanics of material as dynamic bending stress due to its velocity. Blocks are affected by a lot of factors like distribution of applied force on block shape, stiffness and strength of tubes energy absorption of both tubes and foam. under quasi-static test the foam will be compressed while tubes will expose to bending stress. So, moment of area can’t be the only parameter for measuring stress behavior on block but shape dimensions, stiffness, strength, flexural rigidity and the effect of position of reinforcement tube (inner tube) control energy abortion of ACCFBs. The minimum limit for blocks volume is 1/2 to 2/3 inch^3^ according to its shield thickness and stiffness where under this limit *E*_*dd*_ will be reduced by about 20% so if block size needed to be reduced to 1/2 inch^3^ its preferable to use foam twice relative density at least to be between (24 to 30%). This will increase density of foam and mass of block and also increase heat insulation capacity of foam. So, the selection of block volume to 1inch^3^ is optimum.

**Figure 8:**
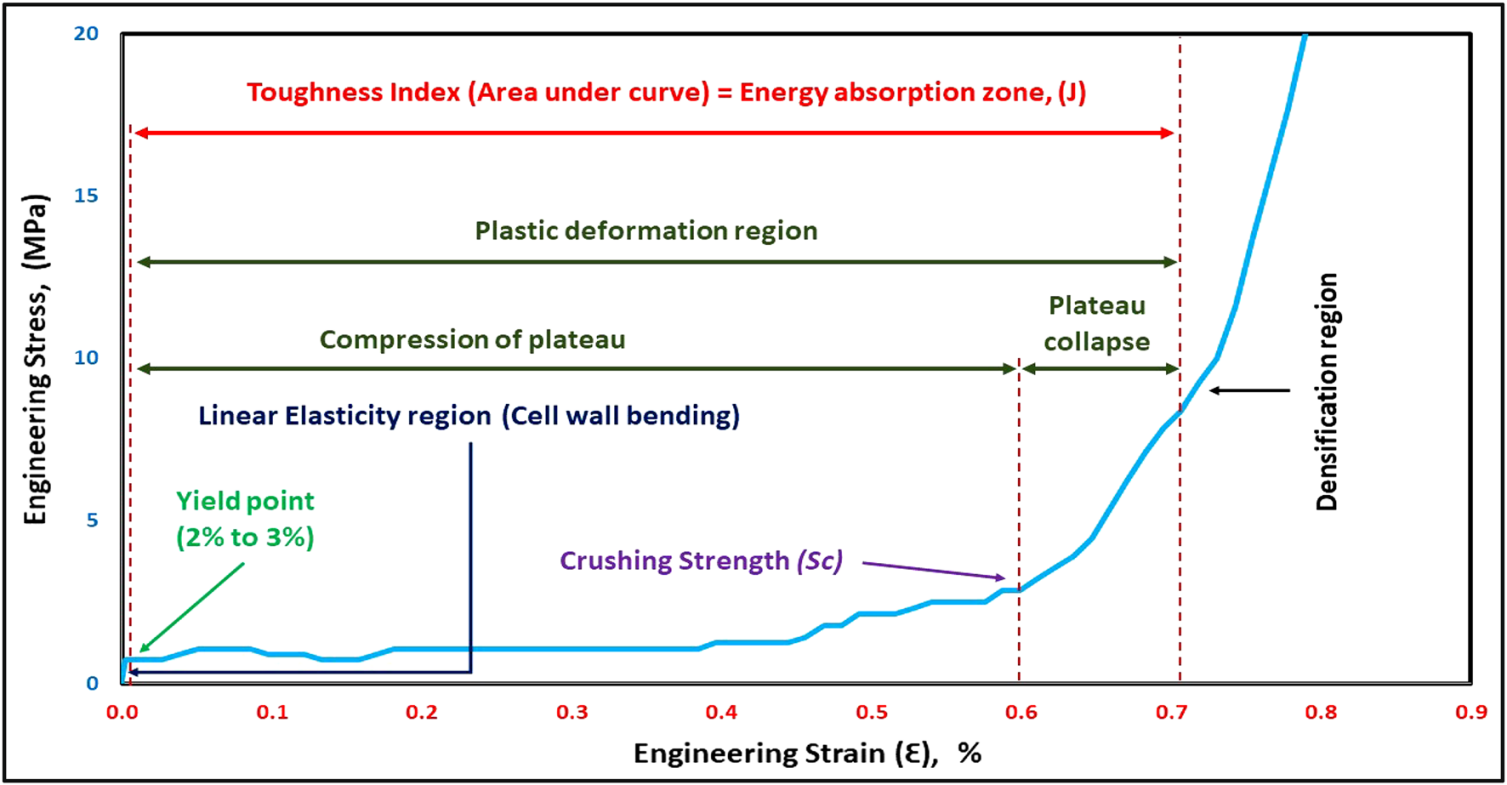
Stress–strain curve regions of Aluminium foam.

The Aluminum foam compression curve below exposes the stress–strain curve regions as appears in Fig. 8*.* Plateau collapse region could be specified through (*S*_*c*_) by applying Eq. (4) or (5). The compressive stress–strain curve of metal foam has three main regions respectively: linear elastic region, plastic region where plateau collapse at the end of it, and densification region where foam density increases due to the full destruction of foam cells. The first region (linear elastic zone) occurred at a small strain (2–3%). The second region (plastic deformation) continues till about 70% strain. The third region (densification) will continue until the solid state^23^.

## Results and discussion

### Compression test of pure Al foam block *(Pure—Sq.)* at strain 70%

**Figure 9:**
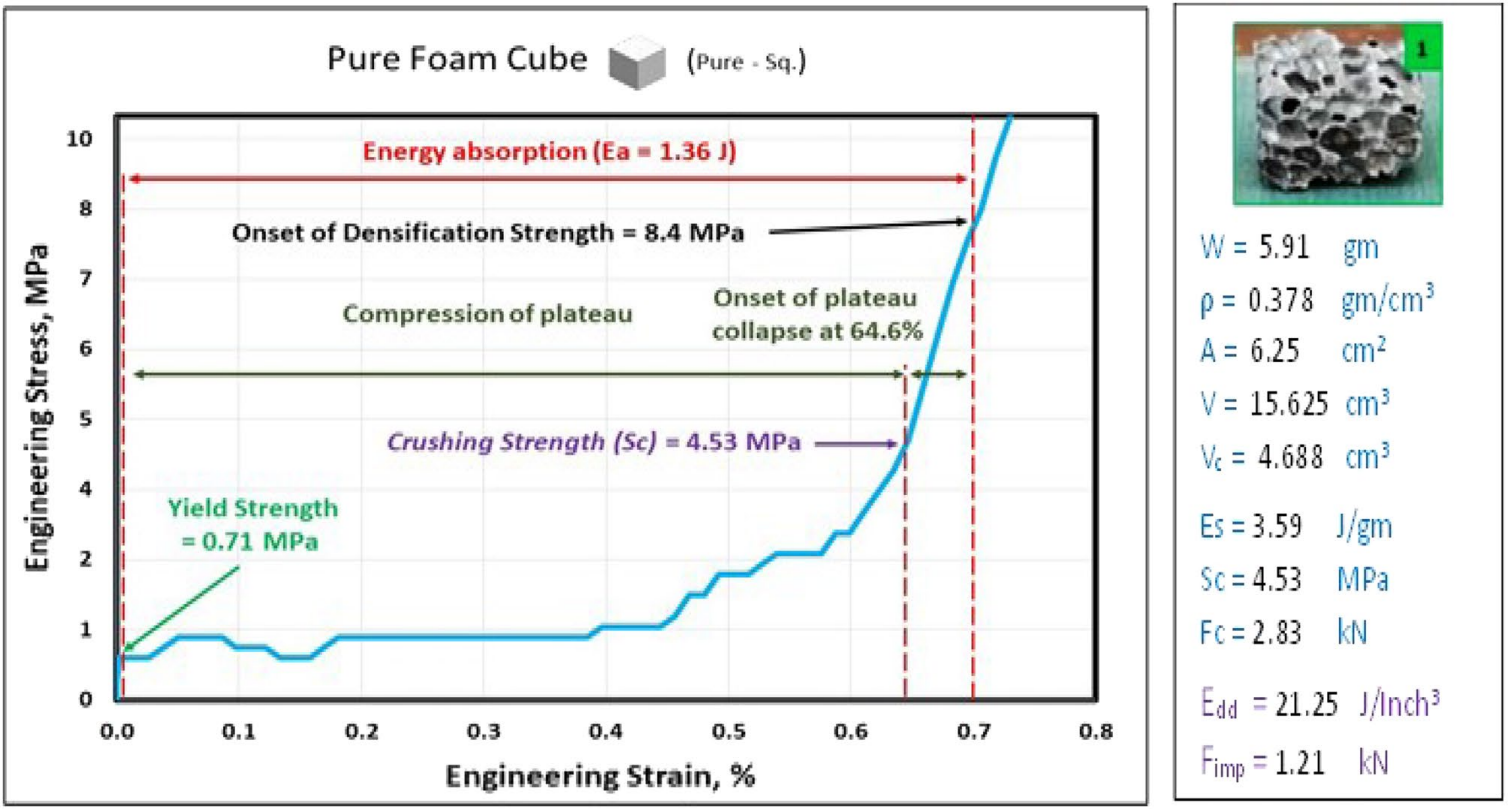
Stress–strain curve of pure aluminium foam block (Pure—Sq.).

Figure 9 shows the engineering stress–strain curve of pure aluminum foam cube. It is clear that the yield strength is 0.71 MPa while, compressive strength is 8.4 MPa (at strain 70%) and crushing strength (Sc) is 4.53 MPa (at strain 64.6%) and energy absorption (area of compression under the curve) is *E*_*a*_ = 1.36 J and* E*_*dd*_ = 21.25 J/inch^3^.

### Compression test of finger phalanx blocks at strain 70%

**Figure 10:**
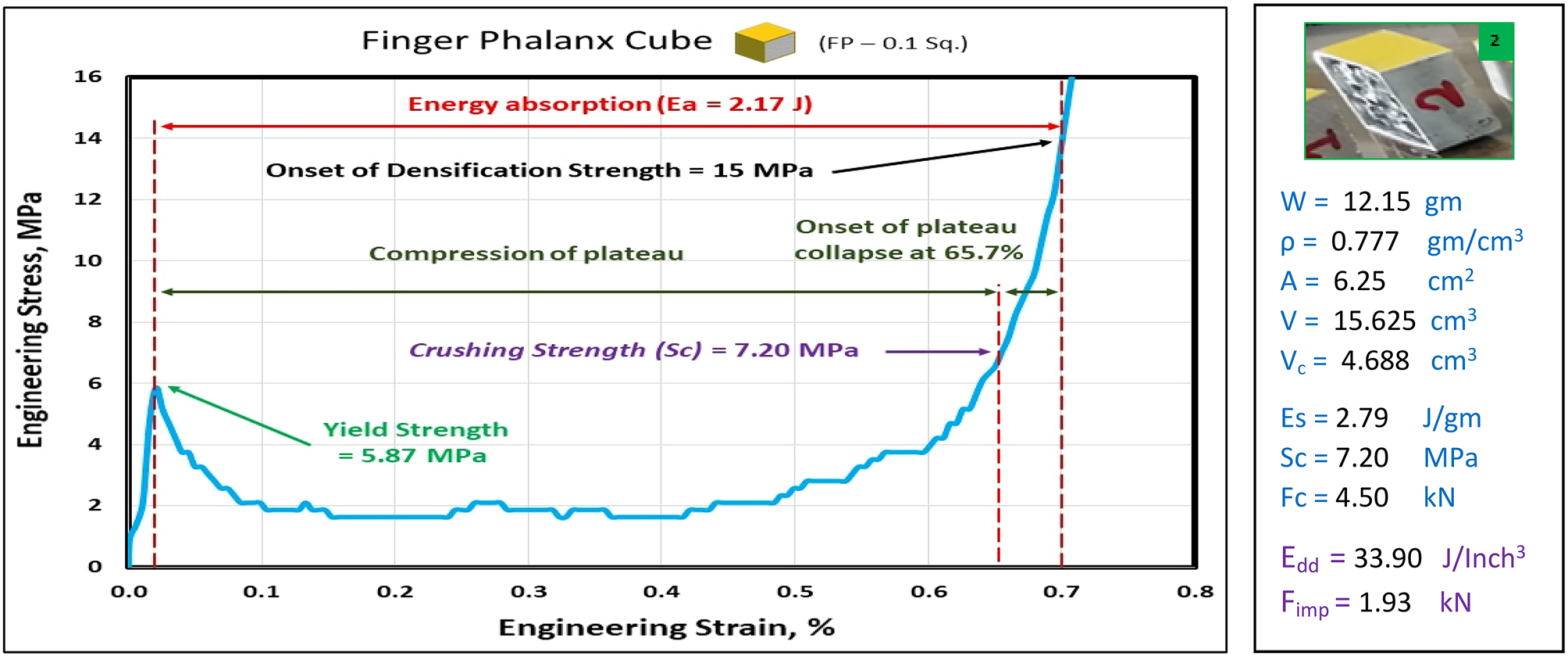
Stress–strain curve of finger phalanx cube block (FP—0.1 Sq.).

**Figure 11:**
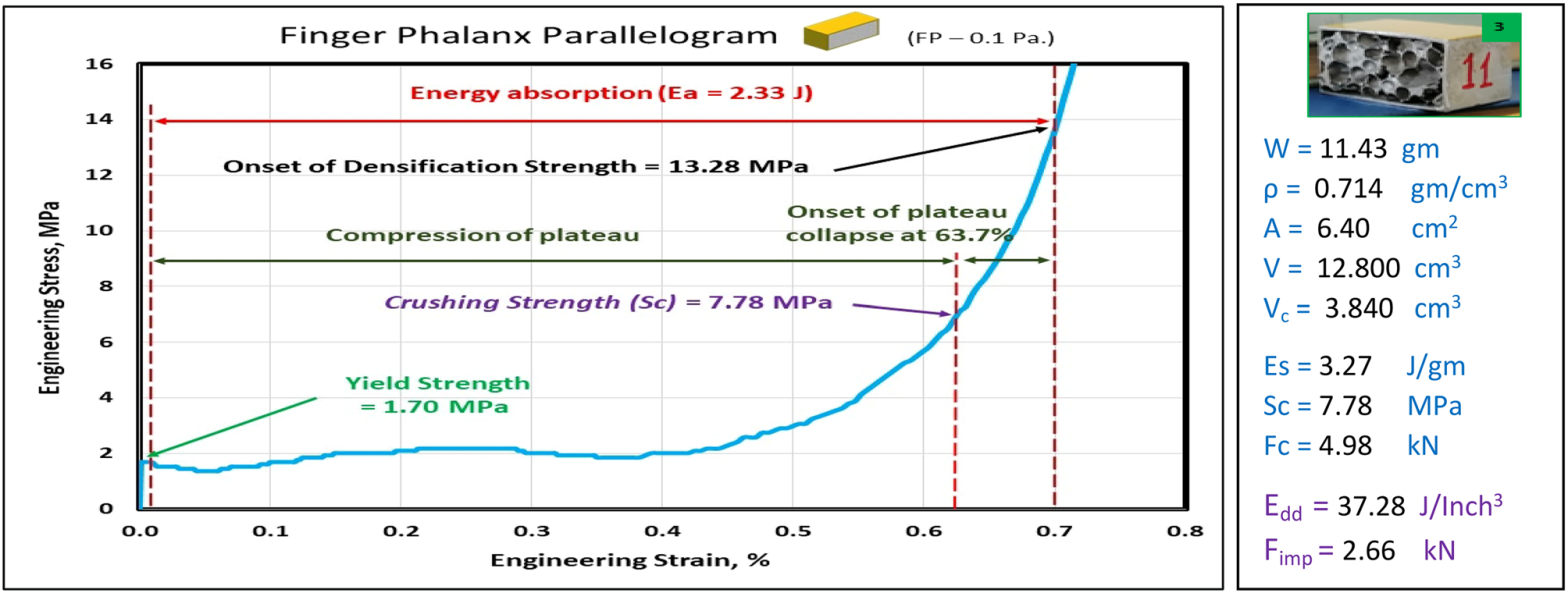
Stress–strain curve of finger phalanx parallelogram block (FP—0.1 Pa.).

**Figure 12:**
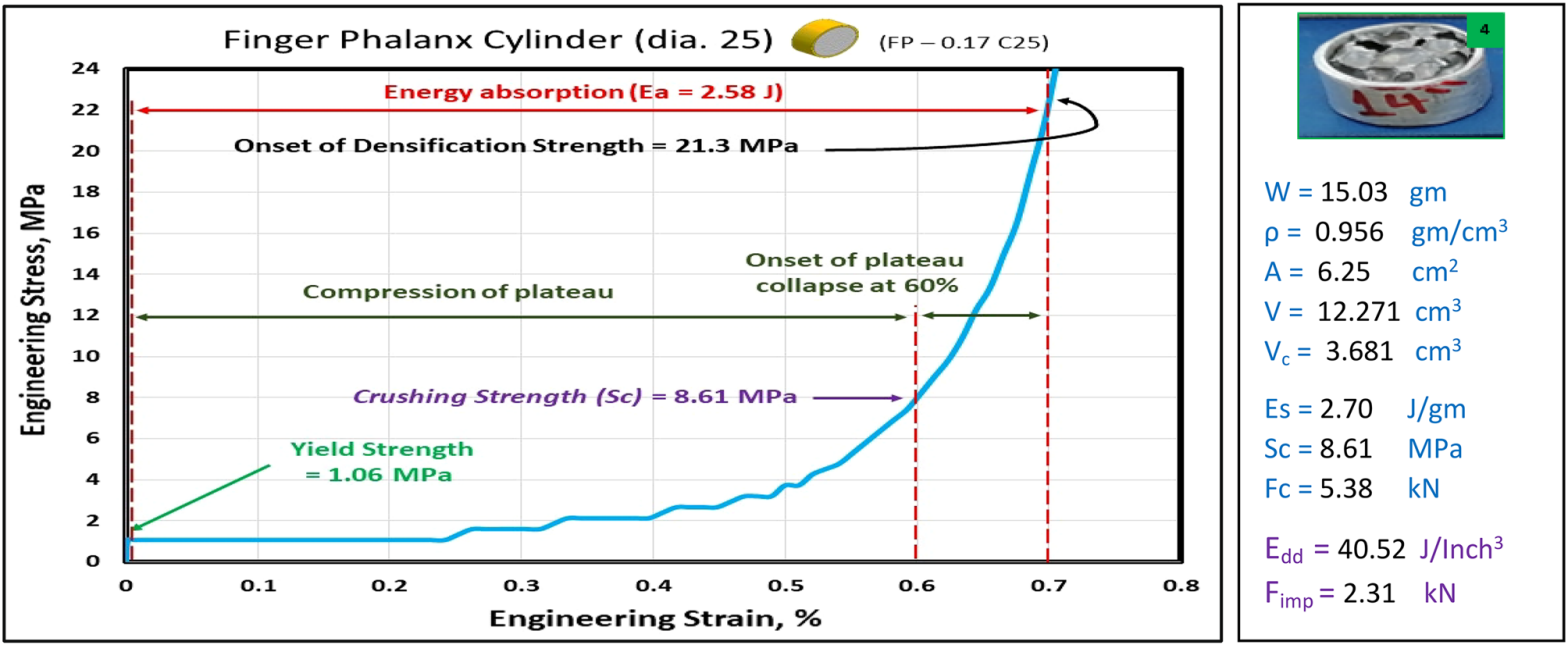
Stress–strain curve of finger phalanx cylinder block (FP—0.17 C25).

**Figure 13:**
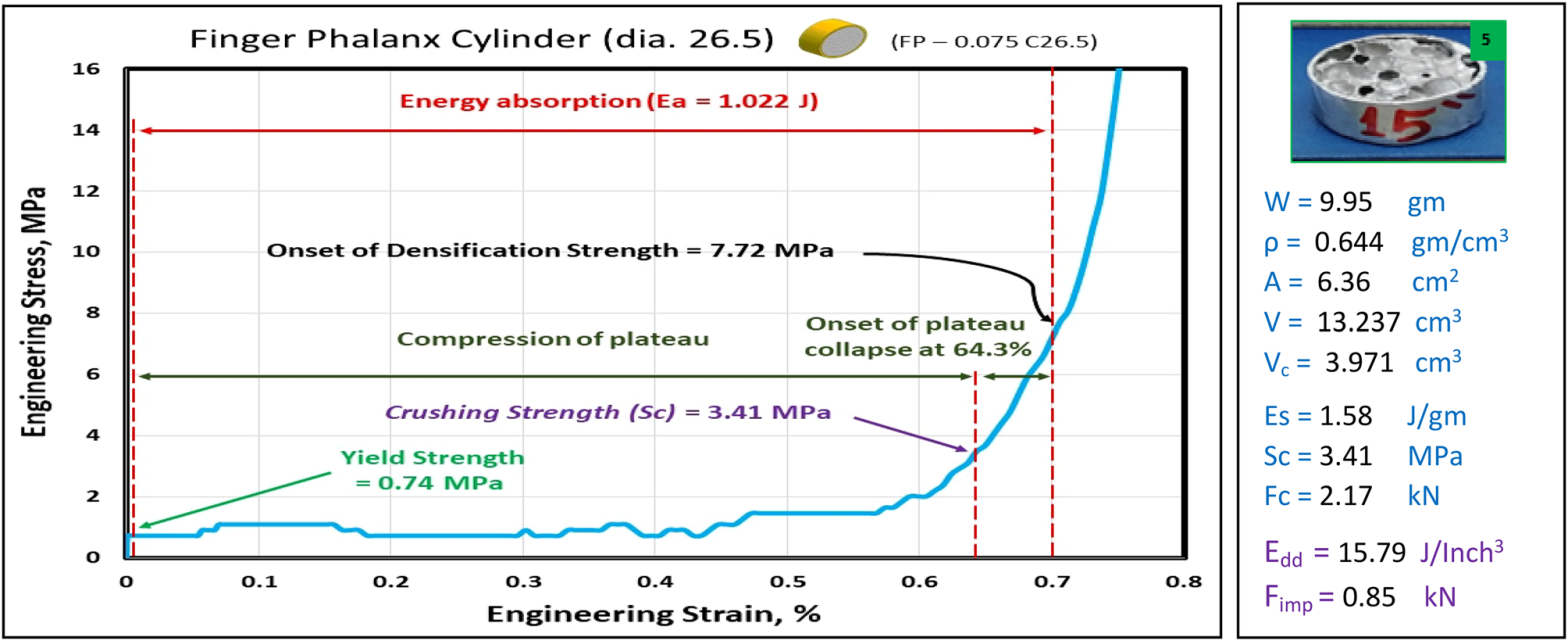
Stress–strain curve of finger phalanx cylinder block (FP—0.075 C26.5).

**Figure 14:**
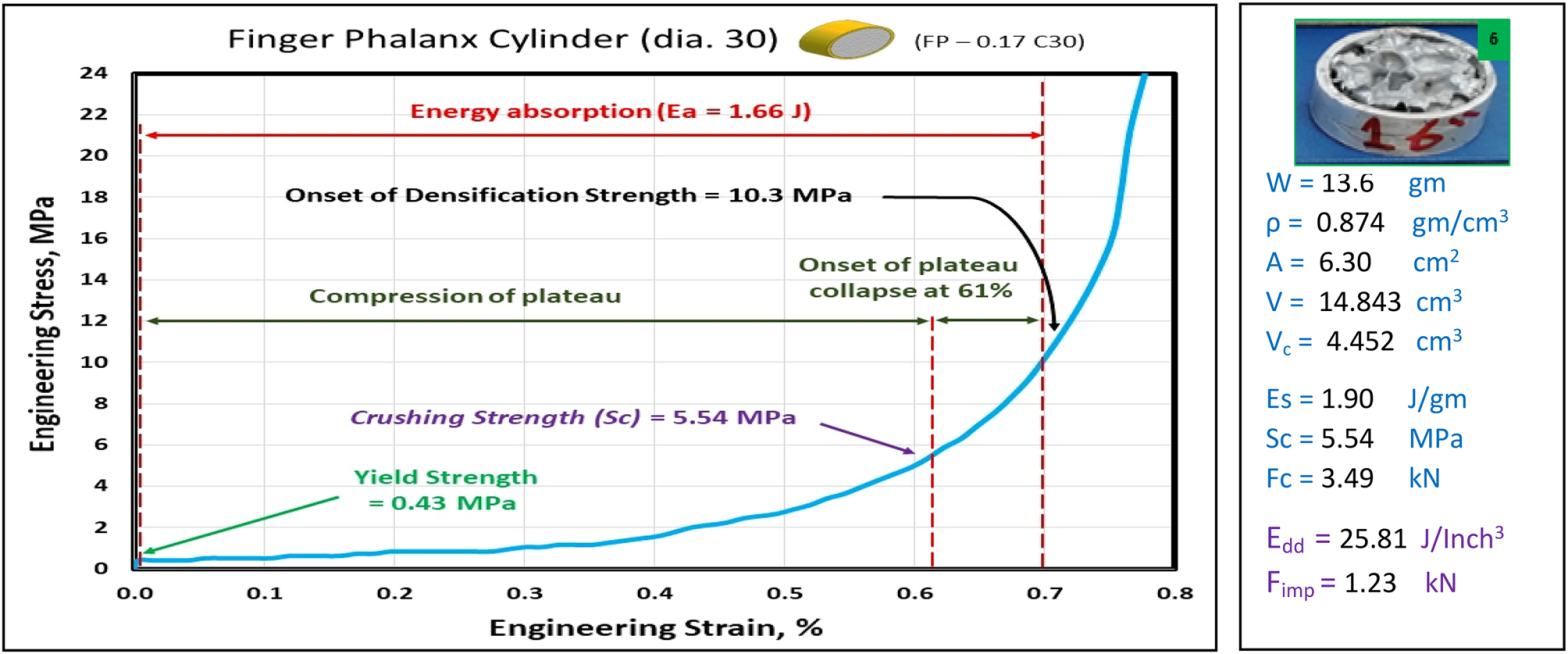
Stress–strain curve of finger phalanx cylinder block (FP—0.17 C30).

Figures 10, 11, 12, 13, and 14 show the engineering stress–strain curve of finger phalanx blocks. It seems clear that the yield strength of the foam cube shielded with square tube (*block: FP—0.1 Sq.*) is the highest value (5.87 MPa) due to the resistance of square shape to deform. while the lowest yield strength is (0.43 MPa) for foam cylinder shielded with circular tube (*block: FP—0.17 C30*). This can be attributed to the large diameter of this shield. Also, it is apparent that the highest crushing strength (*Sc*) is (8.61 MPa) for foam cylinder shielded with circular tube (*block: FP—0.17 C25*) due to the small size of shield and the lowest (*Sc*) is (5.54 MPa) for foam cylinder shielded with circular tube (*block: FP—0.17 C30*) due to large size of shield where the resistance of deformation is reduced, and the collapse process of foam cells dominates that strength depends on cells shapes, wall thickness, size and distribution^24^.

**Figure 15:**
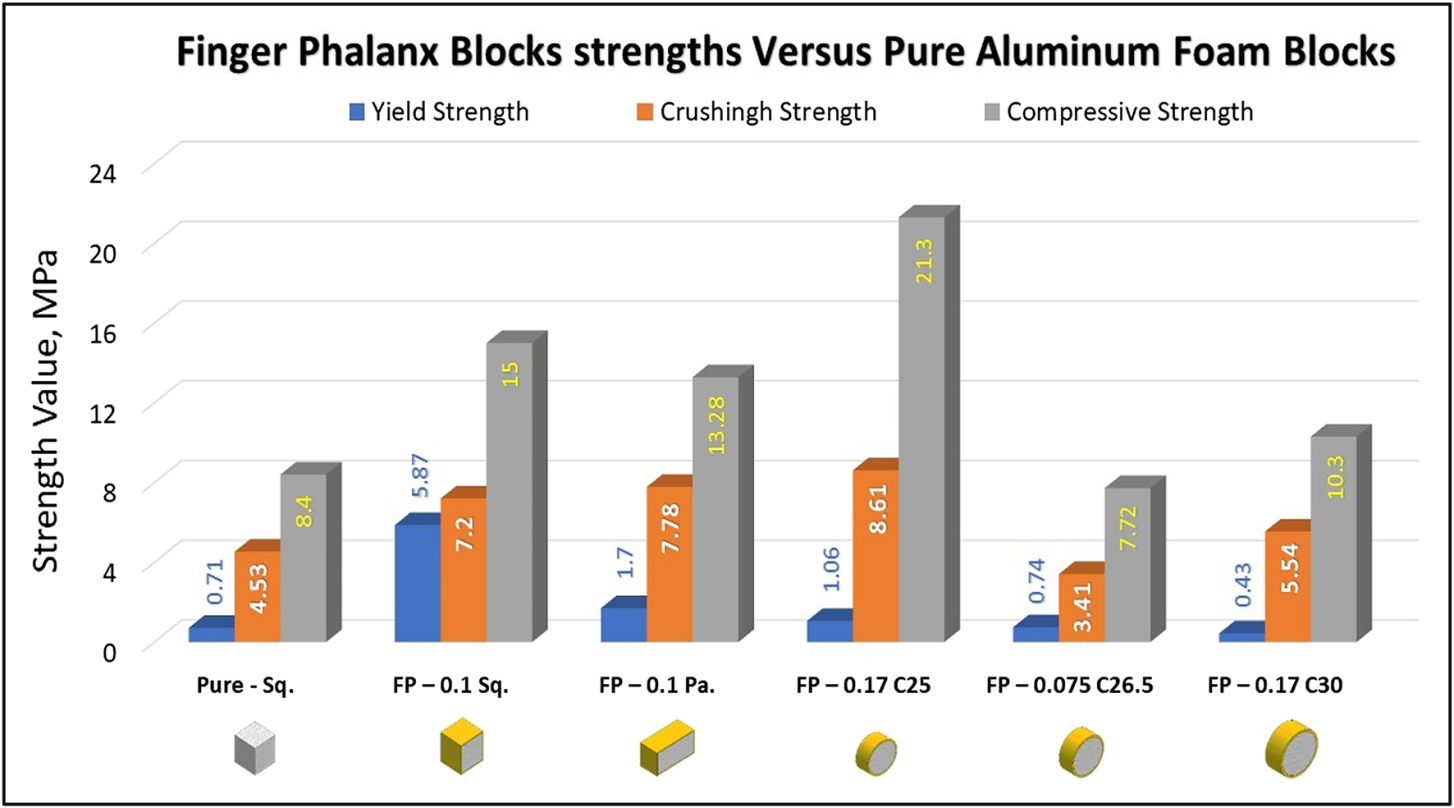
Finger phalanx blocks yield, crushing, and compressive strengths vs Al foam block.

**Figure 16:**
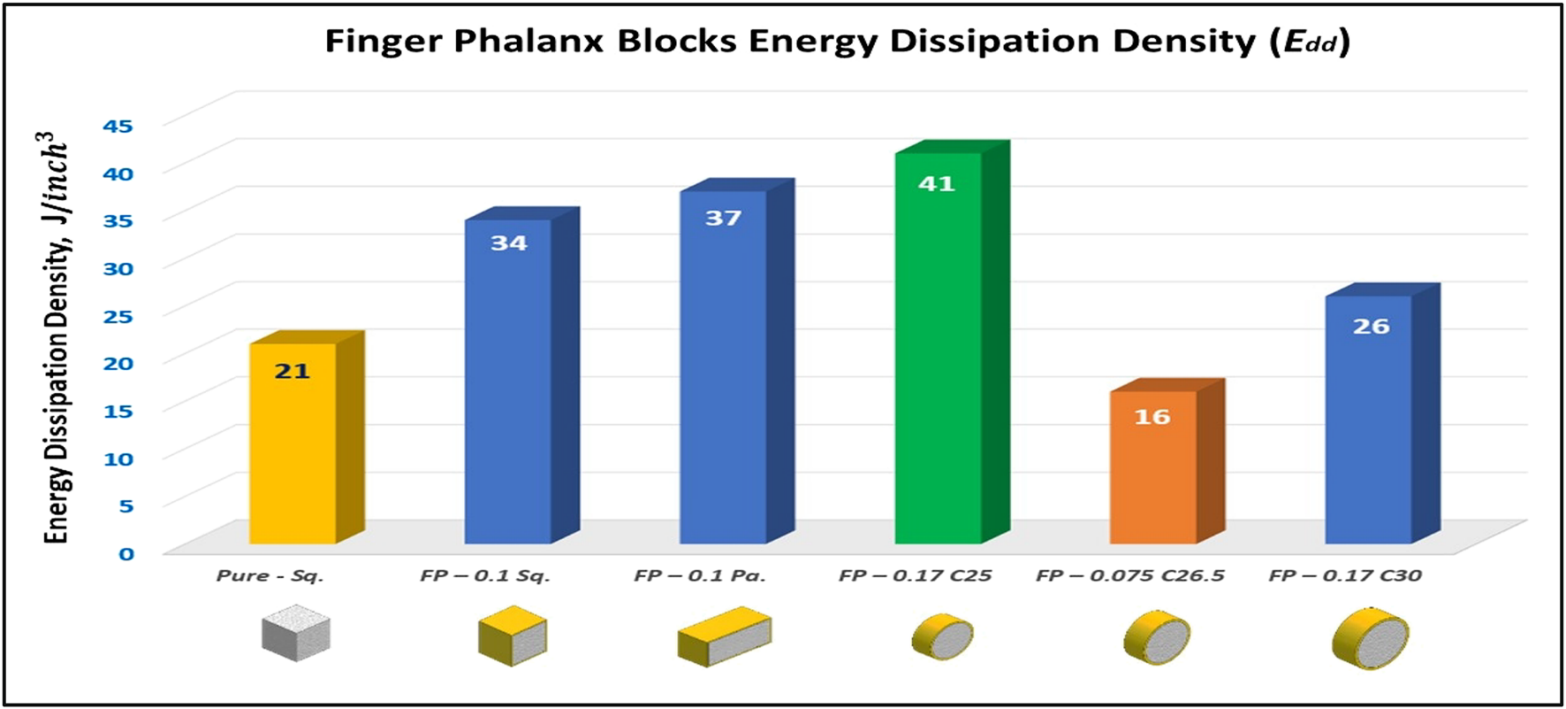
Al foam block and finger phalanx blocks toughness index (total absorbed energy per volume inch^3^).

Figure 15 shows the summary of yield, crushing, and compressive strengths for all finger phalanx blocks by compared with pure aluminum foam blocks. Energy dissipation density (*E*_*dd*_) has been calculated for all finger phalanx blocks at volume 1 inch^3^. Figure 16 shows energy dissipation density (*E*_*dd*_) values for pure Al foam block and finger phalanx blocks with volume 1 inch^3^. Which have been rounded to the nearest whole numbers. This is defined easily as the toughness index for blocks where values have come from energy absorbed which is calculated by area under the curve and then multiplied by volume of the block.

Table 5 exposes the E_dd_ deformation length at strain 70% and energy absorption enhancement percent of blocks versus aluminum foam blocks in, where:

7 $$ Enhancement \, \,percent \, = \left( {E_{dd\,(block)} {-}E_{dd\,(Al)} } \right) \, /E_{dd\,(Al)} $$

**Table 5:** Blocks deformation length at strain 70% and energy absorption enhancement percent.

Block code	Pure-Sq	FP-0.1Sq	FP-0.1 Pa	FP-0.17 C25	FP-0.075 C26.5	FP-0.17 C30
Deformation (mm)	17.5	17.5	14	17.5	18.55	21
E_dd_ (J/ Inch^3^)	21.25	33.9	37.28	40.52	15.79	25.81
Enhancement (%)	Reference	60%	75%	**91%**	**− 26%**	21%

Significant values are in [bold].

Enhancement percent calculations appear that the highest value for block FP-0.17 C25 by 91% and the lowest value for block FP-0.075 C26.5 is less than the pure Al block value by 26%. Results show that energy absorption (E_dd_) is proportional to crushing strength (Sc) while densification strength (S_d_) is proportional to density, shape, dimensions and thickness of shield tubes of blocks. Also, rectangular and circular shape tubes with larger sizes at the same thicknesses will be failure easier than shapes with smaller sizes which need high compression load for failure. Thin wall tubes are able to deform more easily than thick walls for tubes that have the same length^25,26^. So, block FP-0.17 C25 has the highest Sc and the highest S_d_ while block FP-0.075 C26.5 has the lowest Sc and the lowest S_d._

### Compression test of Spine (Vertebrae) blocks at strain 70%

**Figure 17:**
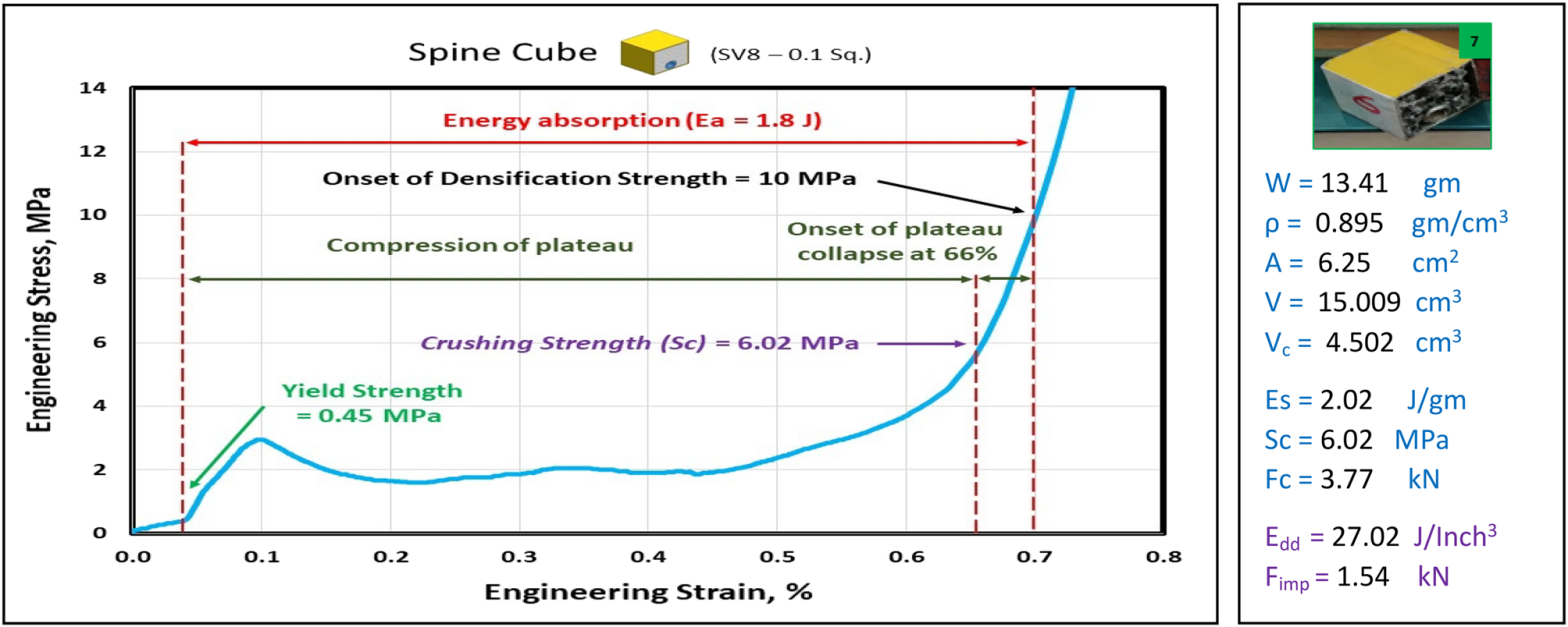
Stress–strain curve of spine cube block (SV8—0.1 Sq.).

**Figure 18:**
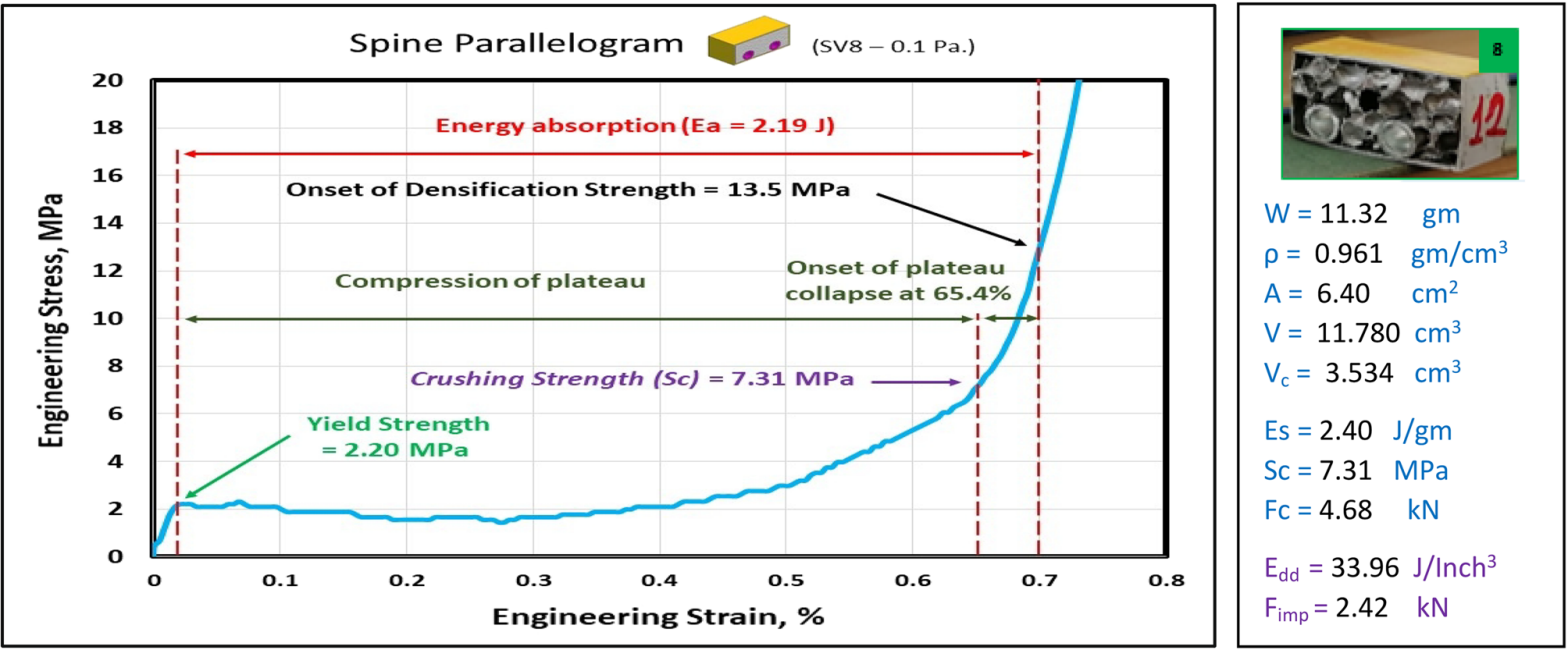
Stress–strain curve of Spine Parallelogram block (SV8—0.1 Pa.).

**Figure 19:**
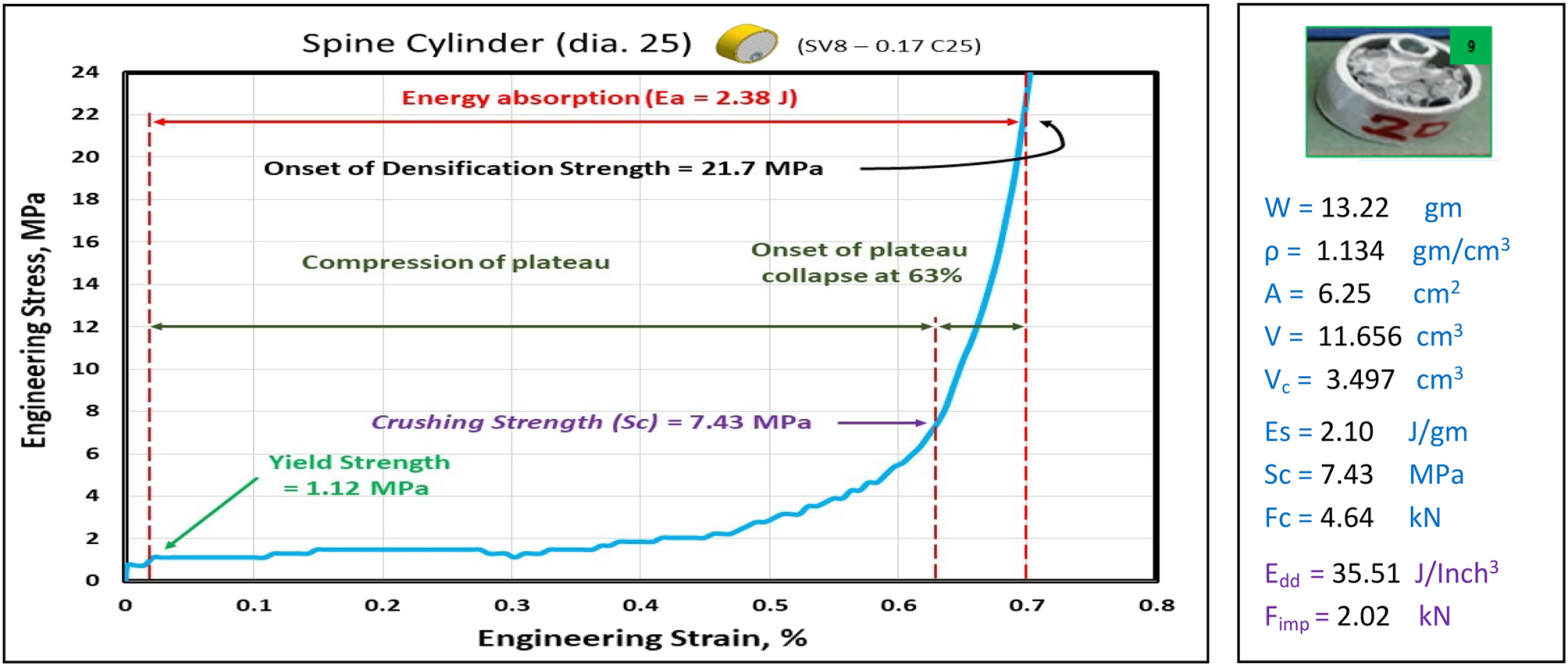
Stress–strain curve of Spine Cylinder block (SV8—0.17 C25).

**Figure 20:**
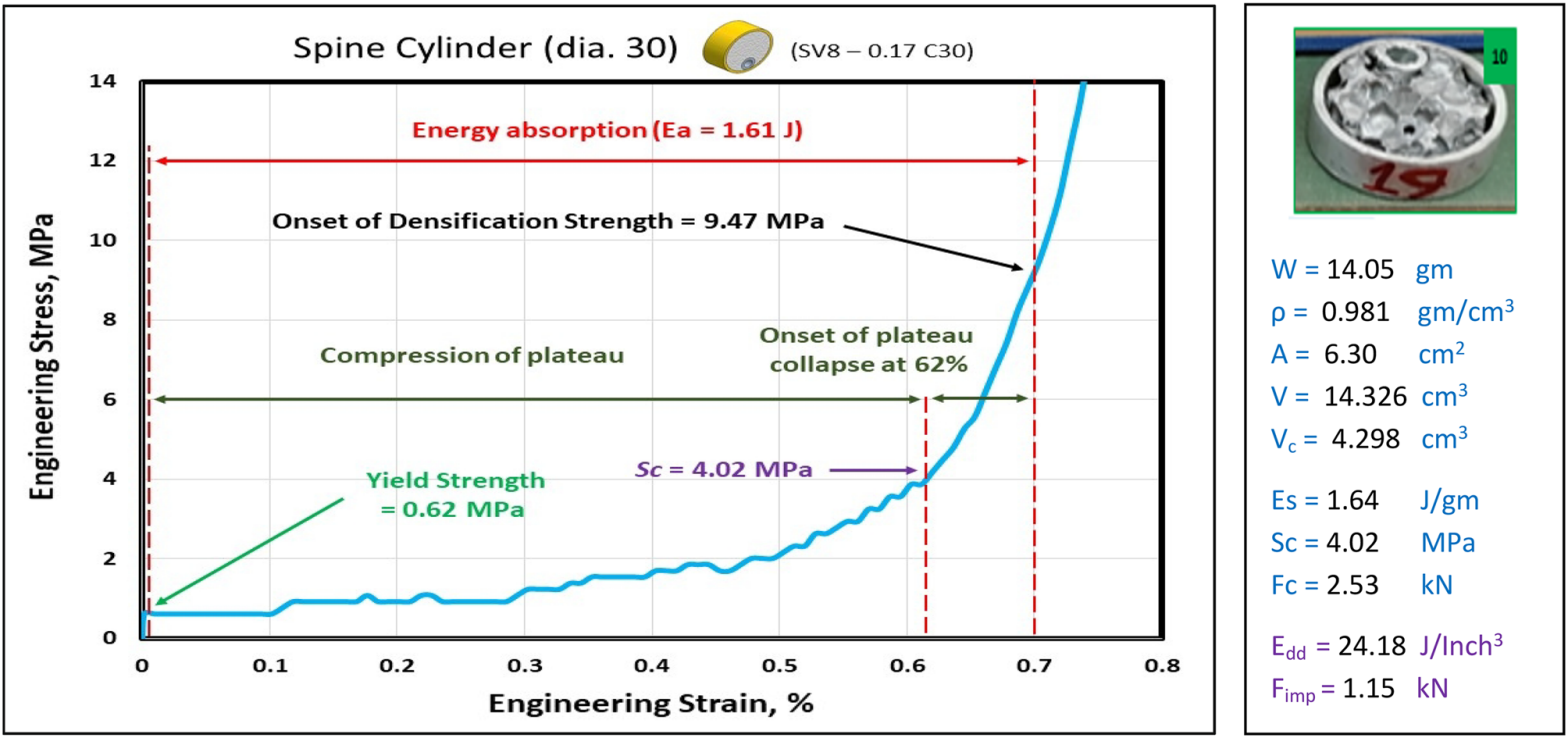
Stress–strain curve of Spine Cylinder block (SV8—0.17 C30).

Figures 17, 18, 19 and 20 show the engineering stress–strain curves of Spine (vertebrae) blocks. It seems clear that the yield strength of foam parallelogram shielded with rectangular tube and containing two offset inner tubes *(block: SV8—0.1 Pa.)* is the highest value (2.2 MPa) due to the resistance of rectangular shape to deform. While the lowest yield strength is (0.45 MPa) for foam cube shielded with square tube and containing one offset inner tube (*block: SV8—0.1 Sq.*). This can be attributed to the low resistance of the foam cube due to its little size and existence of the inner tube. Also, it is apparent that the highest crushing strength (*Sc*)*,* compressive strength and energy absorption of 7.43 MPa, 21.7 MPa and 2.38 J, respectively is for foam cylinder shielded with circular tube (*block: SV8—0.17 C25*) due to the small size of shield and high resistance of inner tube where it compressed till about 20% strain. The lowest (*Sc*), compressive strength and energy absorption of 4.02 MPa, 9.47 MPa and 1.48 J, respectively is for foam cylinder shielded with circular tube (*block: SV8—0.17 C30*) due to the large size of shield where the resistance of deformation is reduced also at strain 70% inner tube will not be compressed. So, this large size will be suitable to guard expensive electrical wires.

**Figure 21:**
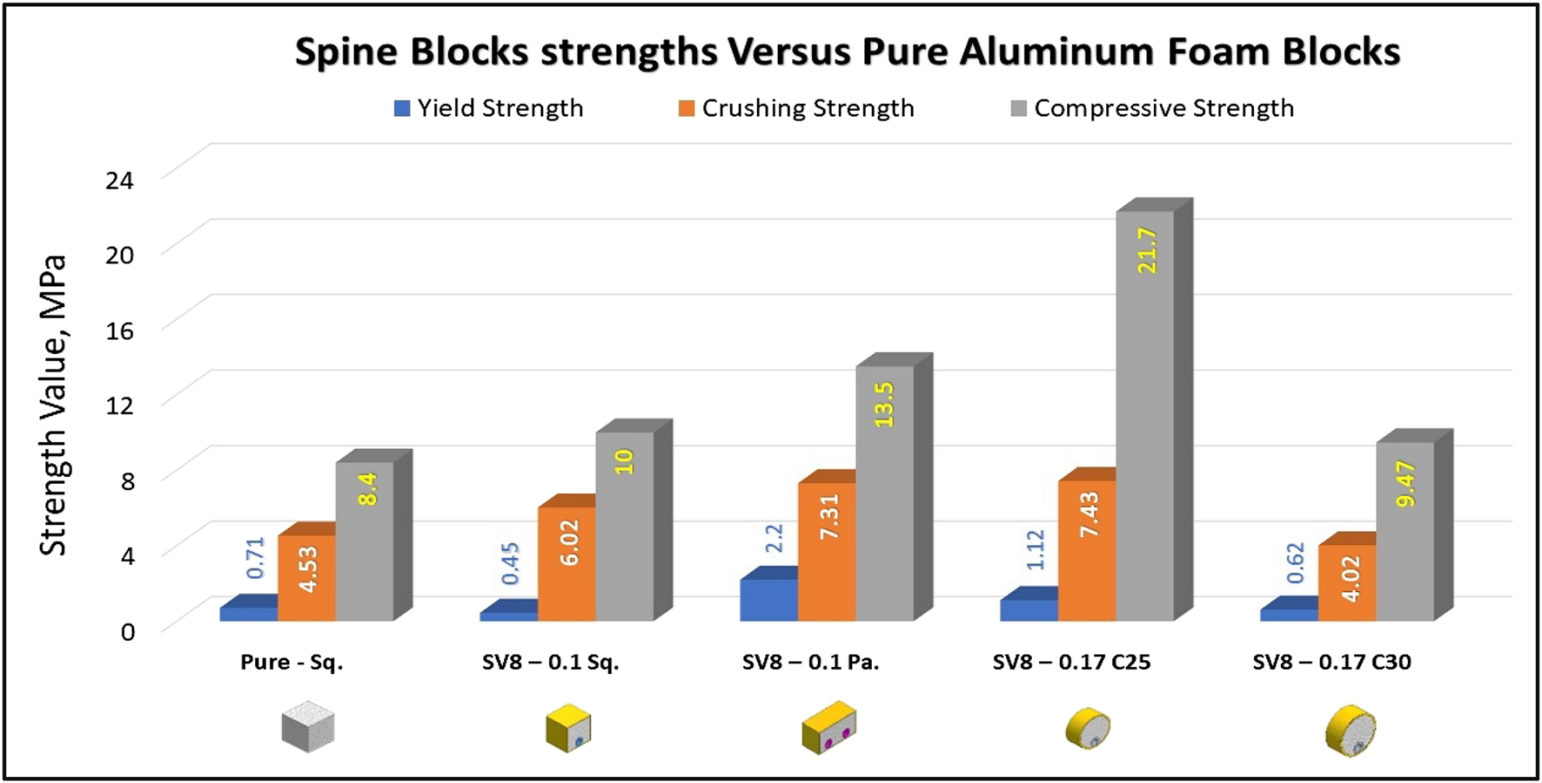
Spine blocks yield, crushing, and compressive strengths vs Al foam block.

**Figure 22:**
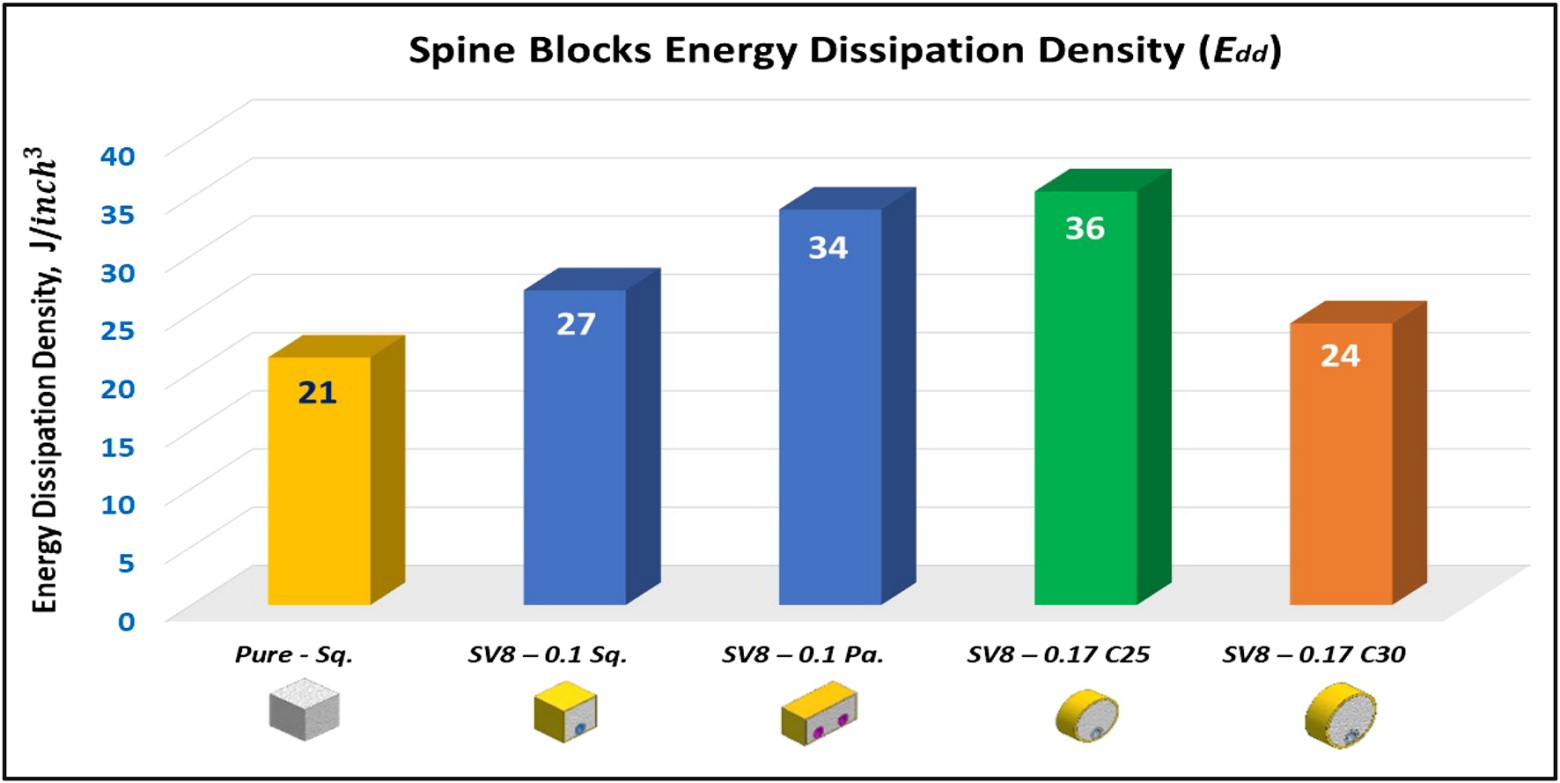
Al foam block and Spine blocks toughness index (total absorbed energy per volume inch^3^).

Figure 21 displays the summary of yield, crushing, and compressive strengths for all spine blocks by compared with pure aluminum foam blocks. Energy dissipation density has been calculated for all Spine blocks at volume 1 inch^3^. Figure 22 shows Energy dissipation density (E_dd_) values for pure Al foam block and Spine blocks with volume 1 inch^3^ which have been rounded to the nearest whole numbers.

Table 6 exposes the E_dd_ with deformation length at strain 70% and enhancement percent of blocks vs aluminum foam block in energy absorption, by applying Eq. (7). Enhancement percent calculations appear that the highest enhancement value for block SV8-0.17 C25 by 67% and the lowest value for block SV8-0.17 C30 by 14%. Results show that E_dd_ is proportional to Sc while densification strength (S_d_) is proportional to shape, dimensions and thickness of shield tubes of blocks. So, block SV8*-0.17 C25* has the highest Sc and the highest S_d_ too while block SV8*-0.17 C30* has the lowest Sc and the lowest S_d_ due to its shield tube large diameter_._ Rectangular block *SV8-0.1 Pa* is the best choice because it has ability to pass two wires through its inner tubes as a saver against impact and has good energy absorption.

**Table 6:** Blocks deformation length at strain 70% and energy absorption enhancement percent.

Block code	Pure-Sq	SV8-0.1Sq	SV8-0.1 Pa	SV8-0.17 C25	SV8-0.17 C30
Deformation (mm)	17.5	17.5	14	17.5	21
E_dd_ (J/ Inch^3^)	21.25	27.02	33.96	35.51	24.18
Enhancement (%)	Reference	27%	60%	**67%**	**14%**

Significant values are in [bold].

### Compression test of Ear canal blocks at strain 70%

**Figure 23:**
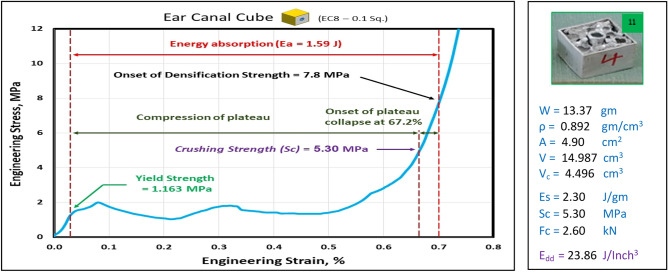
Stress–strain curve of Ear Canal Cube block (EC8—0.1 Sq.).

**Figure 24:**
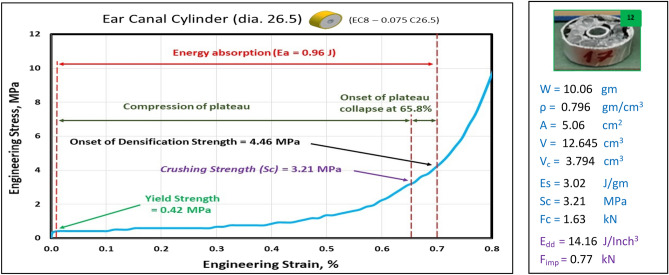
Stress–strain curve of Ear Canal block (EC8—0.075 C26.5).

**Figure 25:**
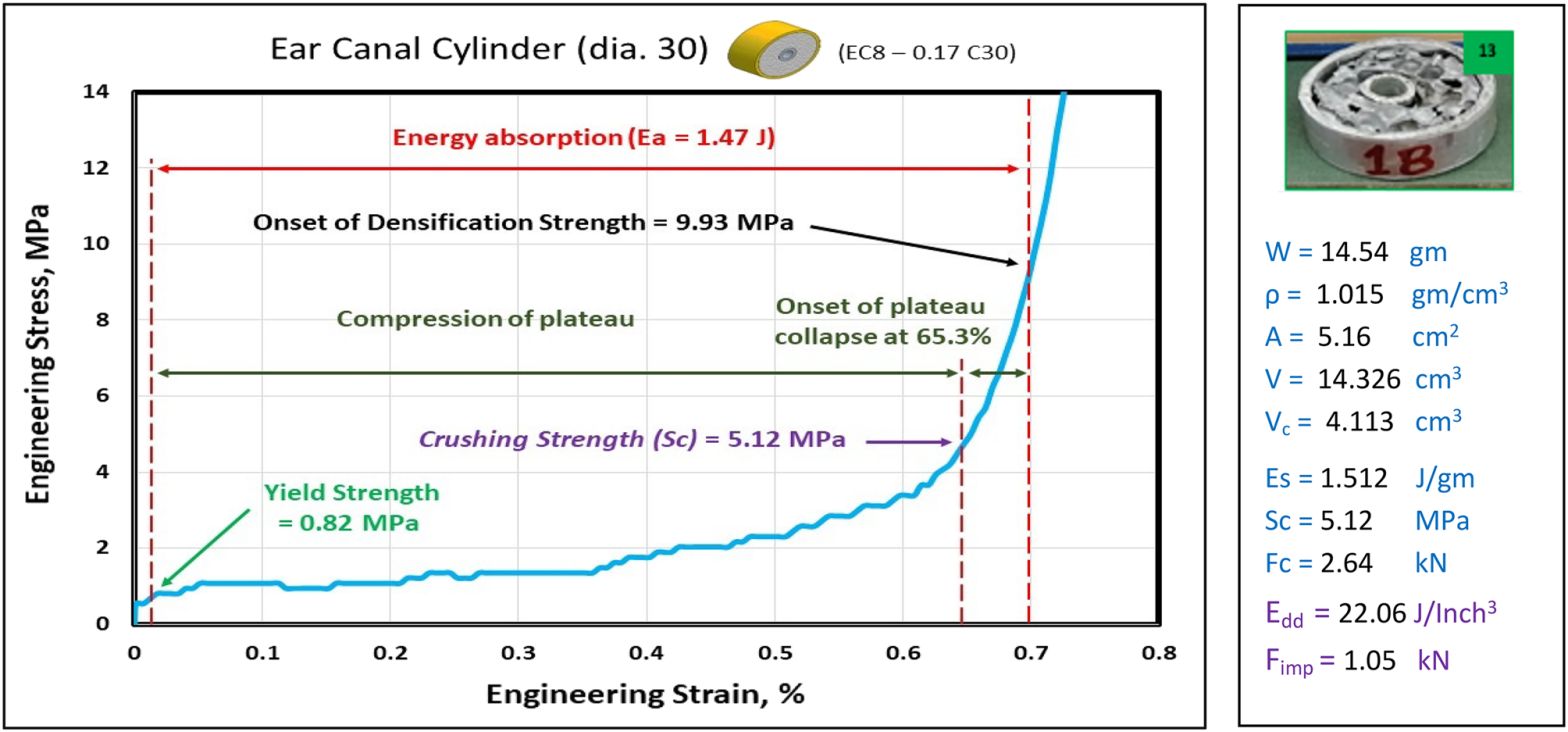
Stress–strain curve of Ear Canal block (EC8—0.17 C30).

Figures 23, 24, and 25 show the engineering stress–strain curve of Ear canal blocks. It seems clear that the yield strength and crushing strength of foam cube shielded with square tubes and containing inner tubes in the center (*block: EC8—0.1 Sq.*) is the highest value of 1.16 and 5.3 MPa, respectively. While the lowest yield strength, crushing strength, compressive strength and energy absorption of 0.42 MPa, 3.21 MPa, 4.46 MPa, and 0.96 J, respectively for foam cylinder shielded with a thin-walled tube which contains inner tube in the center (*block: EC8—0.075 C26.5*). This can be attributed to the low resistance of thin wall tubes and the distribution of foam cells in a cylinder shape with 8 mm pore in the middle which reduces its resistance against compression.

**Figure 26:**
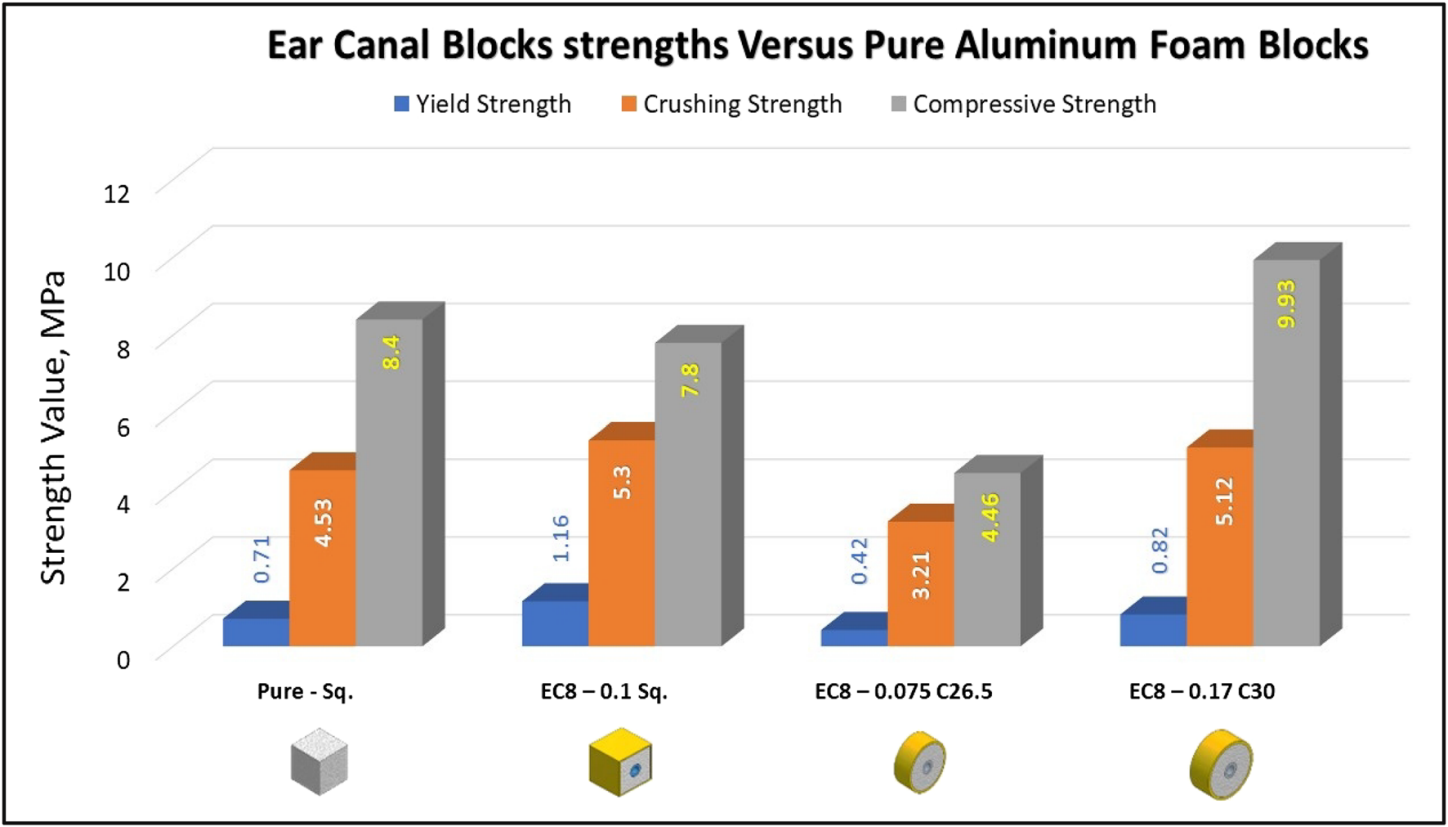
Ear canal blocks yield, crushing, and compressive strengths vs Al foam block.

**Figure 27:**
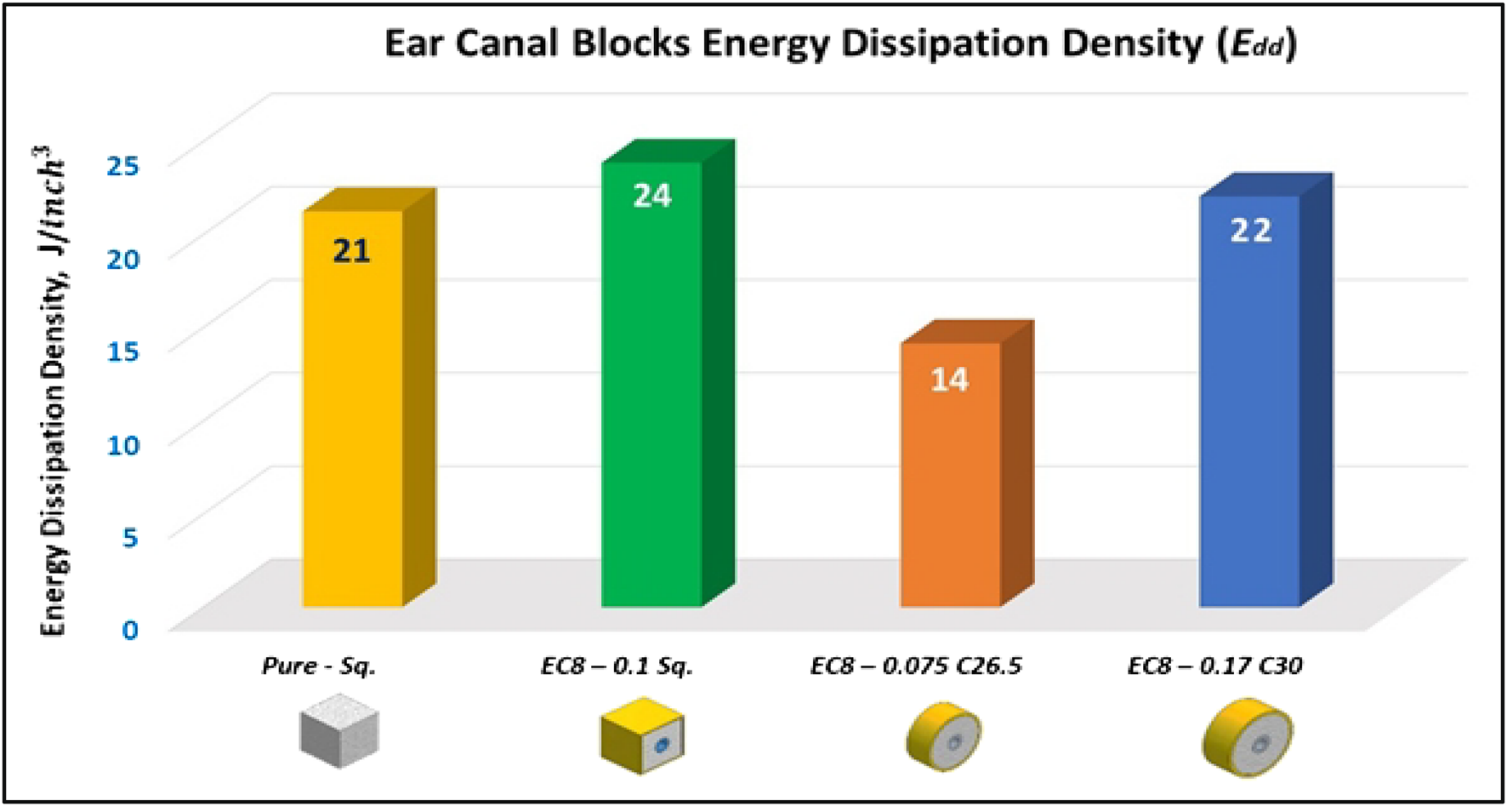
Al foam block and Ear canal blocks toughness index (total absorbed energy per volume inch^3^).

Figure 26 shows the summary of yield, crushing, and compressive strengths for all Ear canal blocks by compared with pure aluminum foam blocks. Figure 27 displays the energy dissipation density (*E*_*dd*_) values for pure Al foam block and Ear canal blocks with volume 1 inch^3^ which have been rounded to the nearest whole numbers.

Table 7 exposes the E_dd_ with deformation length at strain 70% and enhancement percent of blocks vs aluminum foam block in energy absorption, by applying Eq. (7). Enhancement percent calculations appear that the highest enhancement value for block EC8-0.1Sq by 12% and the lowest value for block EC8-0.075 C26.5 is less than pure Al foam by 33%. Results show that E_dd_ is proportional to Sc while densification strength (S_d_) is proportional to shape, dimensions and thickness of shield tubes of blocks. So, block EC8-0.1Sq has the highest Sc and the second arranged for highest S_d_ this refers to its square shape which resists compressive force by uniform projected area this means it needs to increase the load to deform despite it having a thin wall shield so its enhancement percent is relatively little. While block EC8-0.075 C26.5 has the lowest Sc and the lowest S_d_ due to its thin wall shield tube, relatively little length and circular shape.

**Table 7:** Blocks deformation length at strain 70% and energy absorption enhancement percent.

Block code	Pure-Sq	EC8-0.1Sq	EC8-0.075 C26.5	EC8-0.17 C30
Deformation (mm)	17.5	17.5	17.5	21
E_dd_ (J/ Inch^3^)	21.25	23.86	14.16	22.06
Enhancement (%)	Reference	**12%**	**− 33%**	4%

Significant values are in [bold].

Finally, a lot of parameters control the deformation mechanism of blocks under quasi-static test which affects energy absorption values like shield shape, thickness and type of material. Also, foam material, shape and dimensions distribution, cell size, foam density, and foam component and its composition. Simply all aforementioned samples can be concluded in one table to register the energy absorption properties needed for different applications: energy absorption per volume 1 cubic inch (E_dd_) at 70% Strain, and mass. Table 8 exposes properties of all blocks Deformation height at 70% strain, mass and energy absorption for each block.

**Table 8:** All blocks energy dissipation density and impact force results for 1 inch^3^ volume and 70% strain.

Finger phalanx blocks						
Sample code		FP-0.1 Sq	FP-0.1 Pa	FP-0.17 C25	FP-0.075 C26.5	FP-0.17 C30
Weight	(gm)	12.25	11.43	15.03	9.95	13.60
Deformation	(mm)	17.5	14	17.5	18.55	21
E_dd_	(J/Inch^3^)	34	37	41	16	26
Spine (Vertebrae) blocks						

Sample code		SV8-0.1 Sq	SV8-0.1 Pa	SV8-0.17 C25	SV8-0.17 C30	
Weight	(gm)	13.41	14.15	16.88	14.70	
Deformation	(mm)	17.5	14	17.5	21	
E_dd_	(J/Inch^3^)	27	34	36	24	
Ear canal blocks						

Sample code		EC8-0.1 Sq	SV8-0.075 C26.5	EC8-0.17 C30		
Weight	(gm)	13.37	11.74	15.21		
Deformation	(mm)	17.5	18.55	21		
E_dd_	(J/Inch^3^)	24	14	27		

Pure aluminum foam block						
Sample code		Pure—Sq				
Weight	(gm)	5.91				
Deformation	(mm)	17.5				
E_dd_	(J/Inch^3^)	21				

### Applications of ACCFBs

**Figure 28:**
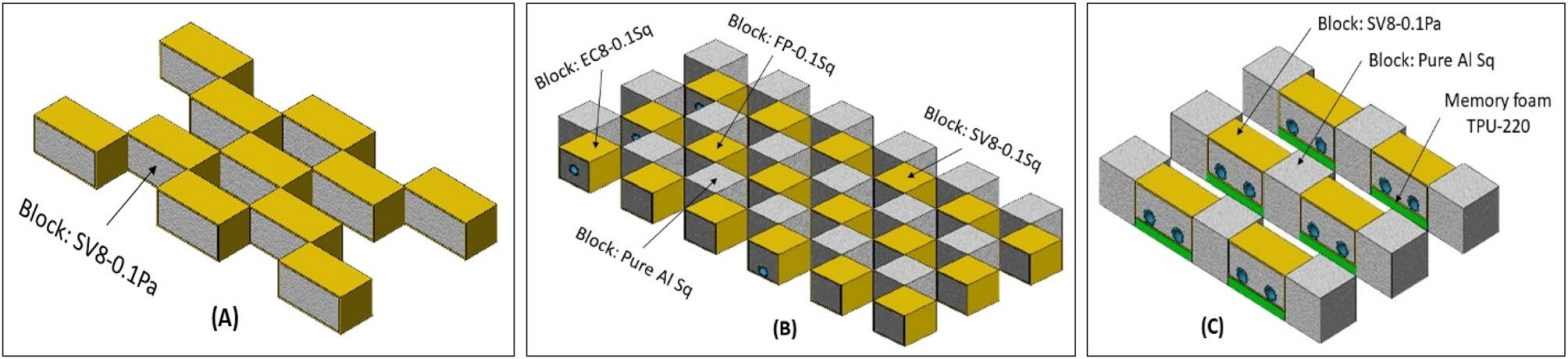
Categories of ACCFBs patterns (**a**) Similar, (**b**) Multi blocks, (**c**) Combined.

The main idea of ACCFBs is their flexibility in use and maintained easily where it is expendable parts can be collected in main three categories as Fig. 28 illustrate (1) Similar blocks pattern which consists of one type of block, (2) Multi blocks pattern consisting of different blocks types, (3) Combined blocks pattern consists of other components plus blocks like memory foam or silicon slices, rubber sleeves or metal canister. Actually, the expansion deformation factor should be considered where shield tubes deformed and expand in width from 115 to 140% according to tube shape while, foam expands in all directions due to impact slightly by about 105%.

The total energy absorption of patterns can be estimated easily at strain 70% but two parameters should be specified besides energy pattern area and total mass. Example of the estimated of total energy absorption of Fig. 28b pattern by referring to Table 8 for calculations in Table 9.

**Table 9:** Calculations of total energy absorption, covered area and mass of pattern (b) in Fig. 28.

Total covered area = (12 × 2.5) × (6 × 2.5) = 450 cm^2^	Height of pattern: 2.5 cm^2^
Pure Al blocks E_dd_ = 18 × 21 = 252 J	Mass = 18 × 5.9 = 106.2 gm
FP-0.1Sq blocks E_dd_ = 12 × 34 = 408 J	Mass = 12 × 12.25 = 147 gm
SV8-0.1Sq blocks E_dd_ = 3 × 27 = 81 J	Mass = 3 × 13.41 = 40.23 gm
EC8-0.1Sq blocks E_dd_ = 3 × 24 = 72 J	Mass = 3 × 13.37 = 40.11 gm
Total pattern E_dd_ = 813 J	Total pattern mass = 333.5 gm

The results of the total energy absorption of pattern are 813 J if all blocks have been replaced with one part of aluminium foam will reduce this area to half but actually, it will give total E_dd_ = 756 J and mass will reduce by about 35% than pattern. Seriously there are some challenges faced aluminium foam panels in applications where foam is good insulator for heat also, can’t pass cables or wire through it and this gives limitation on use big parts of foam in vehicles especially for wide areas will need high amount of panels furthermore it valid to receive impacts in one plan this mean to cover two plans (i.e., XZ, YZ) or oblique impact will need two surfaces which will reflect on amount of needed foam. Also, foam as solid (not flexible) part not suit nonuniform shapes which lead to involved in expensive process like casting or accurate machining.

**Figure 29:**
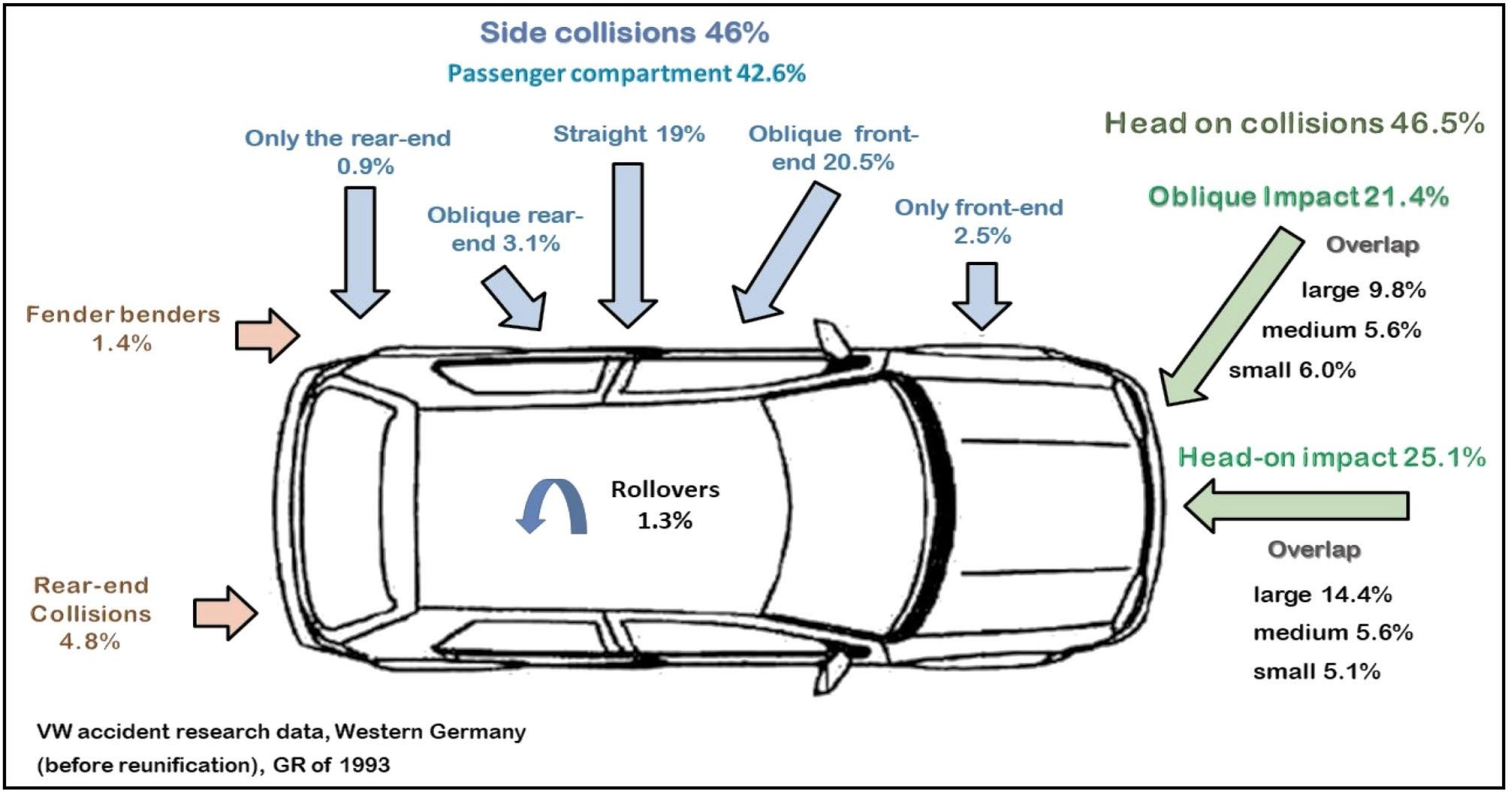
Evaluation of the collision types by Volkswagen company presented on 1993.

**Figure 30:**
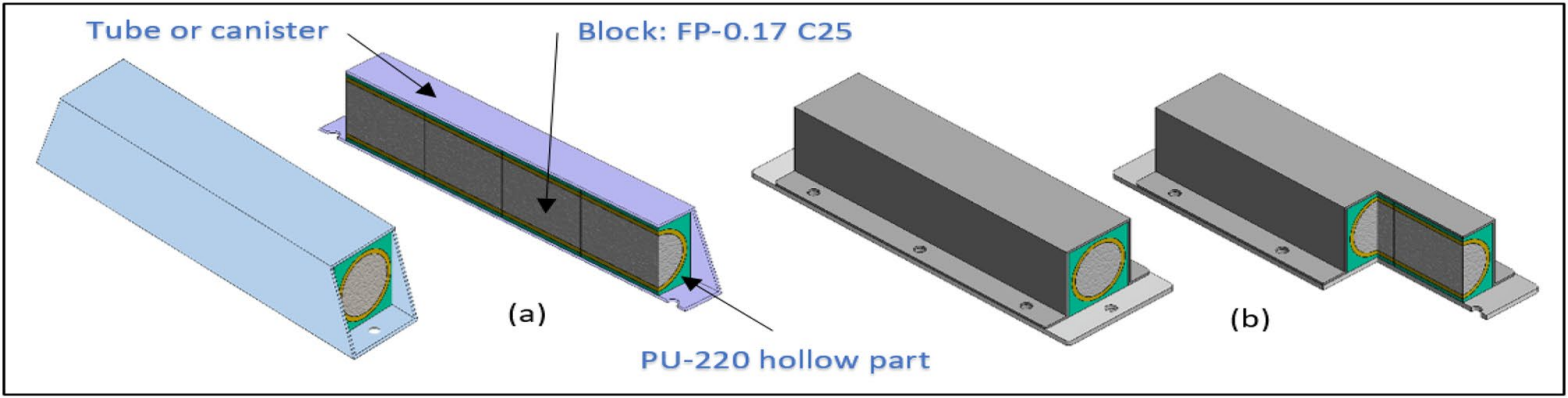
Filled tubes by blocks (**a**) one part squared tube, (**b**) Two parts squared tube (C shape + flat strut).

**Figure 31:**
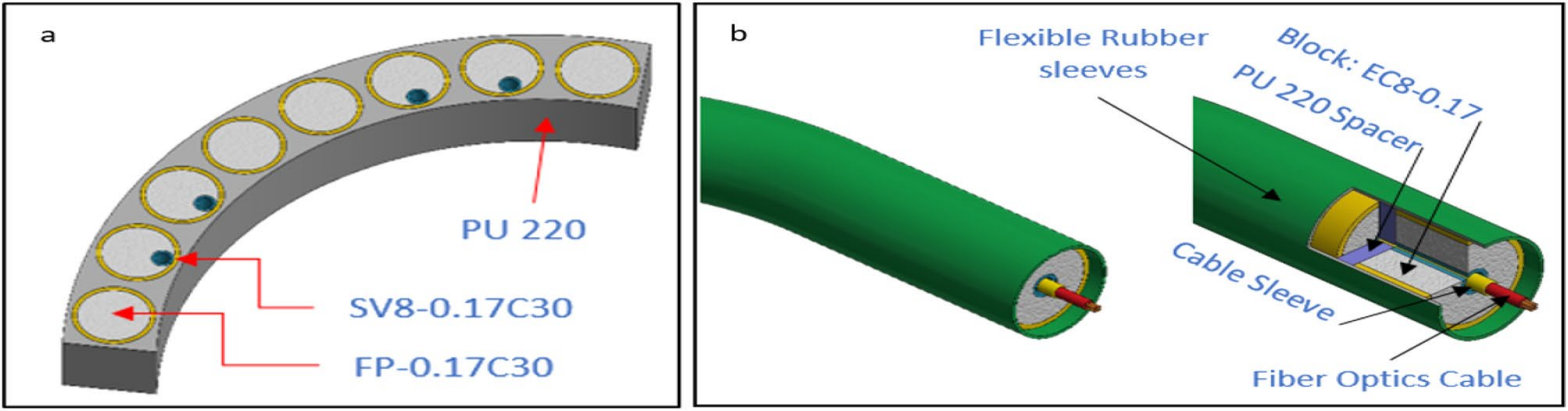
Flexible pattern (**a**) Strand for nonuniform shapes, (**b**) flex shield for guarding wires like fibre optic.

**Figure 32:**
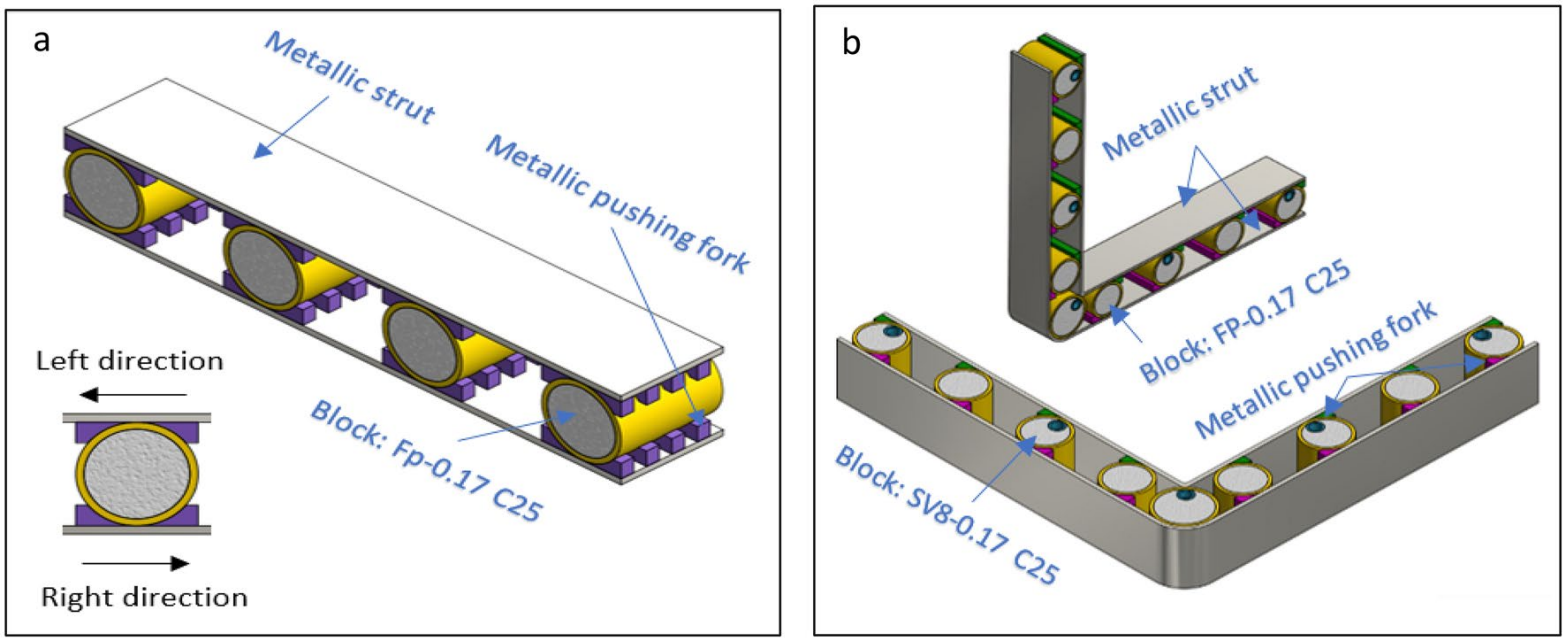
**(a)** Pattern work in two directions **(b)** Pattern work in two plans.

Figure 29 exposes the Evaluation of the collision types and its percentage^27^. Figures 30, 31, 32 illustrates the developed types of combined patterns by research team where polyurethane memory foam (PU-220) with density 220 kg/m^3^ has been used as cushioning and energy absorber material. The peak cushioning efficiency properties at quasi static test were E_dd_ = 0.13 J/cm^3^ (2 J/in^3^), strain 57%, compressive stress was 0.44 MPa, 39% energy return^28^. It is designed to suit most vehicles with considering the aforementioned challenges.

Figure 30 exposes examples of two patterns for filled tubes which depend on the use of highest value of energy absorption for finger phalanx block (FP-0.17 C25) with PU-220 as cushioning material to make slight damping and absorbing energy and make block easy in and easy out in fixation. After fixing the block in the PU-220 which has square shape with segment length of 28 mm block will put 4 parts with total length 128 mm in canister or tube with dimensions 160 × 30 × 30 mm^3^ and thickness 1 mm. A lot of different shapes of canisters or tubes which can consist of 1 part tube or 2 parts assembled together. Length of tube and numbers of used blocks and PU-220 shield could be selected according to required length or application. This filled tube part is suitable for car’s sills front and rear bumpers, frames of buses and pickup cars. It is easy for common man to produce it and fix it. It is easy to calculate the total energy absorption, mass and area of this part after design. This filled tube E_dd_ equals twice E_dd_ of pure Al foam.

Figure 31 displays examples of two flexible patterns one for nonuniform shapes and one for guarding expensive wires. The first one is designed to be able to take any profile shape and different blocks can be used also can cover by belt strands or adhesive with chain strands for heavy applications. The second which guard wires like fibre optic bending angle could be controlled through flexible spacer from PU-220 or soft silicon through using maximum strain for estimate bending angle also cover material stretching should be calculated to avoid passing critical bending angle.

Figure 32 displays examples of two double-motion effect patterns the first one is designed to treat with oblique impact where it is able to work in two directions left and right with high efficiency in using all blocks ability to absorb energy. The second one is designed to improve the efficiency of energy absorption by absorbing energy in two perpendicular planes. They are suitable for protected vehicles with big mass (i.e., pickup cars and trucks) or slow vehicles for heavy work and susceptible to overturn or collisions due to their work in limited areas like forklifts. Patterns can be supported with additional blocks in their spaces but it will work in one direction or one plan also, cooling shouldn’t be considered for equipment where more foam means more heat isolation.

Finally, the ACCFBs properties can be tailored through material selection for metallic foam and shield tubes dimensions and density but should take into consideration for blocks and patterns corrosion potentials of materials to avoid corrosion, heat transfer, working medium and the best distribution for blocks in pattern to take maximum benefits for mass, energy absorption and covered area.

## Conclusions

- Aluminum foam blocks energy absorption has been enhanced due to shielding it by traditional aluminum foam tubes.
- Inner tube diameter should not exceed 1/3 height or diameter of the block to save foam properties as leading properties for energy absorption.
- Block selected to be with optimum designed volume 1 cubic inch to suit all types of foam also to be easy in comparing with other blocks dimensions and energy absorption.
- Each block has its energy absorption and impact force value which give variety for use in different applications, especially for vehicles.
- The compressive strength is directly proportional to the energy absorption.
- The highest value for energy absorption belongs to thick wall cylinder shapes with small diameters (25 mm, t = 1.7 mm) while the lowest value is belonging thin wall cylinder shapes (26.5 mm, t = 0.75 mm) which is less than Al foam cube by about 30%.
- The lowest impact force belongs to thin wall circular shapes and the highest belongs to the finger phalanx parallelogram block. The best properties belonging the circular thick wall blocks with large diameter (30 mm, t = 1.7) where it has high energy absorption and relatively low impact energy.
- Impact force can be used as index for selecting blocks where block has high impact force can be used as a guard for expensive wires like spine and ear canal blocks while block has low impact force will be suitable to absorb energy by high amount which suits for armoring against impacts.

## Data Availability

All data generated or analyzed during this study are included in this published article.
